# Reliability of human retina organoid generation from hiPSC-derived neuroepithelial cysts

**DOI:** 10.3389/fncel.2023.1166641

**Published:** 2023-10-06

**Authors:** Madalena Carido, Manuela Völkner, Lisa Maria Steinheuer, Felix Wagner, Thomas Kurth, Natalie Dumler, Selen Ulusoy, Stephanie Wieneke, Anabel Villanueva Norniella, Cristina Golfieri, Shahryar Khattak, Bruno Schönfelder, Maria Scamozzi, Katja Zoschke, Sebastian Canzler, Jörg Hackermüller, Marius Ader, Mike O. Karl

**Affiliations:** ^1^Center for Regenerative Therapies Dresden (CRTD), TU Dresden, Dresden, Germany; ^2^German Center for Neurodegenerative Diseases (DZNE) Dresden, Dresden, Germany; ^3^Department Computational Biology, Helmholtz Centre for Environmental Research—UFZ, Leipzig, Germany; ^4^Department of Computer Science, Leipzig University, Leipzig, Germany; ^5^Center for Molecular and Cellular Bioengineering (CMCB), Technology Platform, Core Facility Electron Microscopy and Histology, TU Dresden, Dresden, Germany; ^6^Center for Molecular and Cellular Bioengineering (CMCB), Stem Cell Engineering Facility, TU Dresden, Dresden, Germany

**Keywords:** organoid, human, retina, hiPSC, stem cells, neurodevelopment, pathology, gliosis

## Abstract

The possible applications for human retinal organoids (HROs) derived from human induced pluripotent stem cells (hiPSC) rely on the robustness and transferability of the methodology for their generation. Standardized strategies and parameters to effectively assess, compare, and optimize organoid protocols are starting to be established, but are not yet complete. To advance this, we explored the efficiency and reliability of a differentiation method, called CYST protocol, that facilitates retina generation by forming neuroepithelial cysts from hiPSC clusters. Here, we tested seven different hiPSC lines which reproducibly generated HROs. Histological and ultrastructural analyses indicate that HRO differentiation and maturation are regulated. The different hiPSC lines appeared to be a larger source of variance than experimental rounds. Although previous reports have shown that HROs in several other protocols contain a rather low number of cones, HROs from the CYST protocol are consistently richer in cones and with a comparable ratio of cones, rods, and Müller glia. To provide further insight into HRO cell composition, we studied single cell RNA sequencing data and applied CaSTLe, a transfer learning approach. Additionally, we devised a potential strategy to systematically evaluate different organoid protocols side-by-side through parallel differentiation from the same hiPSC batches: In an explorative study, the CYST protocol was compared to a conceptually different protocol based on the formation of cell aggregates from single hiPSCs. Comparing four hiPSC lines showed that both protocols reproduced key characteristics of retinal epithelial structure and cell composition, but the CYST protocol provided a higher HRO yield. So far, our data suggest that CYST-derived HROs remained stable up to at least day 200, while single hiPSC-derived HROs showed spontaneous pathologic changes by day 200. Overall, our data provide insights into the efficiency, reproducibility, and stability of the CYST protocol for generating HROs, which will be useful for further optimizing organoid systems, as well as for basic and translational research applications.

## Introduction

Since the discovery of embryonic and induced pluripotent stem cells and the realization of the potential they offer in regenerative medicine, researchers have focused on the development of defined protocols for efficiently producing multiple cell populations and 3D organ-like tissues, so called organoids. Retina organoids were among the first (Eiraku et al., 2011; Meyer et al., 2011; Nakano et al., 2012). The retina is a complex stratified neuroepithelium composed of seven major cell types: Cone and rod photoreceptors in the outer nuclear layer (ONL); bipolar, horizontal, and amacrine interneurons together with Müller glia cell bodies in the inner nuclear layer (INL); and retinal ganglion cells in the innermost layer, connecting the retina to higher brain centers. Since the early studies (Meyer et al., 2011; Nakano et al., 2012; Zhong et al., 2014), several different protocols have been developed for human retina organoids (HROs) (Llonch et al., 2018; Wagstaff et al., 2021). Some protocols have already been further optimized and used for diverse applications, like pathology modeling and preclinical studies advancing gene- or cell-based therapies. Studies of the human primary retina have provided a wealth of information (Hoshino et al., 2017; Sridhar et al., 2020; Eldred and Reh, 2021; Singh et al., 2021; Hussey et al., 2022; Kim et al., 2023) facilitating the validation of organoid systems. However, there is still no systematic understanding of HRO protocol commonalities and differences in organoid development and application potential, and additional strategies for effective comparison studies are needed. So far, organoid protocols and the rationales of application studies differ at multiple levels, which complicates their comparability and thus slows scientific progress. Interestingly, although most protocols so far seem to share the ability to reproduce key aspects of retinal development, there are also major differences. For example, most protocols report organoids that are rod-photoreceptor dominant (e.g., Nakano et al., 2012; Kaewkhaw et al., 2015; Capowski et al., 2019), while fewer report organoids which in comparison are richer in cone photoreceptors (Völkner et al., 2022; Kim et al., 2023; Supplementary Table 1). This might be due to the use of different cell lines, culture conditions, and application of growth factors or other modulators facilitating organoid development. For example, human induced pluripotent stem cell (hiPSC) lines might be different at the genomic level due to different cell donors, tissue sources, and reprogramming methods, and it is still unclear to what extent this affects HRO systems. Another example is conceptional protocol differences, most notably the first step of organoidogenesis from pluripotent cells: Organoid differentiation may start with the production of aggregates from a defined number of dissociated single pluripotent cells (Nakano et al., 2012; Boucherie et al., 2013; Kaewkhaw et al., 2015; Wiley et al., 2016; Wahlin et al., 2017; Welby et al., 2017; Hallam et al., 2018) or from small cell clumps directly produced by dissociation of pluripotent stem cells (Meyer et al., 2011; Zhong et al., 2014; Vergara et al., 2017; Capowski et al., 2019; Kaya et al., 2019; Mellough et al., 2019; Cowan et al., 2020). At least in some protocols it seems still unclear if pluripotent stem cell derived aggregates or clumps differentiate in the different protocols into retina via an embryoid body (EB)-like state or directly via the neuroectodermal lineage. EBs are 3D cell aggregates capable of producing all germ layers (Hopfl et al., 2004), and are commonly used in differentiation protocols for generating a wide range of cell lineages. In a different approach, Matrigel-embedded hiPSC clusters spontaneously produce a lumen-containing epithelial cyst that displays apical-basal polarity within 1–2 days with neuroepithelial identity followed by eyefield identity within 5 days (Zhu et al., 2013). Depending on the media conditions, these eyefield epithelia can be differentiated into retinal pigment epithelial cells (Zhu et al., 2013; Almedawar et al., 2020; Schreiter et al., 2020), HROs (Zhu et al., 2013; Lowe et al., 2016; Völkner et al., 2021b,^2022^; Kim et al., 2023), and possibly also other parts of the nervous system, like spinal cord-like organoids (Meinhardt et al., 2014). A better understanding of each of the current protocols, development of further parameters for defined stages of organoidogenesis, and effective strategies to compare protocols to each other may provide the basis for future systematic comparisons (and thus optimization) of organoid systems, including organoid systems effectively tuned for differential applications.

Here, we used several different approaches to study the reliability and robustness of the neuroepithelial cyst-based (CYST) HRO protocol: We assessed organoid development, efficiency, and reproducibility, including major retinal cell type composition, and its variance across up to seven hiPSC lines derived by different methods and from different source cells. We analyzed single cell RNA sequencing data, using manual and automated (CaSTLe, a transfer learning approach) annotation strategies, to gain insight into HRO cell composition. We further explored the efficiency of the CYST protocol using parallel differentiation started to compare it with one of the pioneering and conceptionally different HRO protocols based on aggregates derived from a defined number of dissociated single pluripotent cells. Our data demonstrate that the cyst-based system reliably provides high quality HROs with comparable yields, and a high proportion of cone cells across multiple hiPSC lines: It also provides insights relevant for further organoid system application and optimization.

## Materials and methods

### hiPSC generation and maintenance

All procedures involving hiPSCs were performed in accordance with the ethical standards of institutional and national research committees, as well as with the 1964 Helsinki declaration and its amendments. This research study is part of a project that has been approved by the ethics committee of the TU Dresden (EK390102017). The newly-generated CRTD3 (hPSCreg: CRTDi003-B^1^) hiPSC line (Supplementary Figure 1) and several previously published or commercially available ones (CRTD1, CRTD2, 5A, ND5, GBE, and IMR90) were used in this study (Table 1). The CRTD3 hiPSC line was generated from CD34-positive cells isolated from peripheral blood of consenting healthy donors (ethics committee of Technische Universität Dresden, EK 363112012). CD34-positive cells were reprogrammed using the CytoTune-iPS 2.0 Sendai Reprogramming Kit (Thermo Fischer Scientific, Darmstadt, Germany) using the supplier’s recommendations for transduction. Transduced cells were plated onto hES-qualified Matrigel (Corning, NY, USA) and kept in ReProTeSR medium (Stem Cell Technologies, Cologne, Germany) until colonies were ready to be isolated (18–21 days). Individual hiPSC colonies were mechanically picked, expanded as clonal lines on Matrigel-coated culture dishes in mTeSR1 (Stem Cell Technologies, Cologne, Germany), and adopted to passaging using ReLeSR (Stem Cells Technologies). All hiPSC lines were routinely maintained on Matrigel-coated culture dishes in mTeSR1 and passaged using ReLeSR. Master and working hiPSC banks were generated for the study. To characterize the newly-generated CRTD3 hiPSC line, a series of tests were performed. To analyze the expression of pluripotency markers via flow cytometry the following antibodies were used according to the manufacturer’s instructions: Alexa Fluor 488 anti-OCT3/4 (BD Pharmingen, San Jose, CA, USA), PE anti-SOX2 (BD Pharmingen, San Jose, CA, USA), V450-SSEA-4 (BD Pharmingen, San Jose, CA, USA), and Alexa Fluor 647 anti Tra-1-60 (BD Pharmingen, San Jose, CA, USA). Three-germ-layer differentiation was performed as previously described (Cheung et al., 2011), and characterized by the 3-Germ Layer Immunocytochemistry Kit (Thermo Fisher Scientific, CatNo: A25538) according to the manufacturer’s instructions. SOX17 antibody (Abcam, Cambridge, UK) was used for endoderm identification. Standard G banding karyotyping was performed by the Institute of Human Genetics, University Clinic Jena, Germany.

**TABLE 1:** List of hiPSC lines used for generating HROs.

Acronym	Line name	Origin	Gender	Cell of origin	Reprogramming method	Original publication	Previously used for retinal differentiation
5A	CLN3-iPSC-5A	Collaborator (A. Hermann)	Male	Skin fibroblasts	Retroviral transfection (OCT4, SOX2, KLF4, cMYC)	Lojewski et al., 2014	Völkner et al., 2022
CRTD1	CRTD1 hiPSC	CRTD iPS facility	Male	Foreskin fibroblasts	Non-integrating Sendai virus (OCT3/4, SOX2, KLF4, cMYC)	Völkner et al., 2022	Völkner et al., 2022
CRTD2	CRTD2 hiPSC	CRTD iPS facility	Female	CD34 + peripheral blood	Non-integrating Sendai virus (OCT3/4, SOX2, KLF4, cMYC)	Völkner et al., 2022	Völkner et al., 2022
CRTD3	CRTD3 hiPSC	CRTD iPS facility	Female	CD34 + peripheral blood	Non-integrating Sendai virus (OCT3/4, SOX2, KLF4, cMYC)	This study	–
ND5	ND41865*C	NHCDR, NINDS	Male	Fibroblasts	Retroviral transfection (OCT4, SOX2, KLF4, cMYC)	Almeida et al., 2012	–
GBE	Gibco episomal hiPSC	Thermo Fisher	Female	CD34 + cord blood progenitors	Episomal expression (OCT4, SOX2, KLF4, cMYC, NANOG, LIN28, SV40L T)	Burridge et al., 2011	Zhong et al., 2014; Vergara et al., 2017; Luo et al., 2018
IMR90	IPS (IMR90)-4	WiCell	Female	Fetal lung fibroblasts	Lentiviral transfection (OCT4, SOX2, NANOG, LIN28)	Yu et al., 2007	Meyer et al., 2009; Phillips et al., 2012; Zhong et al., 2014; Gonzalez-Cordero et al., 2017; Vergara et al., 2017; Wahlin et al., 2017; Ovando-Roche et al., 2018; Völkner et al., 2022

### Human retinal organoidogenesis

Human retina organoids (HROs) were generated using either the cyst-based (CYST) (Zhu et al., 2013; Lowe et al., 2016; Völkner et al., 2022) or aggregate-based (AGG) protocol (Nakano et al., 2012), both modified from previously published work (Supplementary Figures 4A, B).

*CYST protocol* – Undifferentiated hiPSCs (60–80% confluency) were passaged to small cell clusters using ReLeSR, resuspended in growth factor-reduced Matrigel (Corning, NY, USA, 354230) on ice and then placed at room temperature (RT) for gelification. Matrigel was gently dispersed by pipetting into small clumps and cultured floating in 6-well low-attachment plates (Nunclon Sphera, Thermo Fisher) in N2B27 medium [1:1 DMEM/F12 GlutaMAX: Neurobasal A medium, 1% B27 with Vitamin A (Thermo Fisher), 0.5% N2 (Gibco), 1% pen/strep (penicillin/streptomycin, Gibco), 0.5% L-GlutaMAX (Gibco), 0.1mM ß-mercaptoethanol (Carl Roth, Karlsruhe, Germany)]. hiPSC-derived cell clusters spontaneously formed neuroepithelial cysts with a single central lumen within the first 3 days. On day (D) 5, cysts were plated onto Matrigel (Corning, NY, USA, 354230) -coated 6-well plates for adherent cell culture conditions. On D13, adherent cultures showed expanded epithelial tissues that were detached intact using Dispase (Stem Cell Technologies, Cologne, Germany) and transferred to floating culture conditions in B27 medium [DMEM/F12 GlutaMAX, 2% B27 without Vitamin A (Gibco), 1% pen/strep, 1% NEAA (non-essential amino acids, Gibco), 0.1% amphotericin B (Gibco)] in 10 cm ultra low-attachment culture dishes (Corning, NY, USA). Retinal epithelial evaginations were manually isolated between D24 and D31 using surgical tweezers (Dumont No. 5) under microscope vision. From D25, 10% FBS (fetal bovine serum, Gibco) was added to the B27 medium. On D100, cultures were changed to N2 FBS medium (DMEM/F12 GlutaMAX, 1% N2, 10% FBS, 1% pen/strep, 0.1% amphotericin B). Synthetic retinoid analogue EC23 (0.3 μM, Tocris, Wiesbaden-Nordenstadt, Germany) was supplemented from D25 to D120. Media was changed every 2–3 days.

*AGG protocol* – Undifferentiated hiPSCs (60–80% confluency) were dissociated to a single-cell suspension using TrypLE Express (Gibco) and resuspended in retinal differentiation medium [RDM; GMEM, 1% NEAA, 1% sodium pyruvate, 1% pen/strep, 1.5% KSR (knock-out serum replacement, Gibco), 0.1mM ß-mercaptoethanol], supplemented with 3 μM Wnt inhibitor (IWR1e, Biomol, Hamburg, Germany) and 20 μM ROCK inhibitor (Y-27632, Stem Cell Technologies, Cologne, Germany). Cells (9,000/well) were seeded onto lipidure-coated 96-well V-bottom plates (Nunclon, NOF Corporation, Tokio, Japan), leading to the formation of one 3D cell aggregate per well. On D2, 2% growth factor-reduced Matrigel was added to the culture medium and, at D12, all cell aggregates from one 96-well plate were transferred to a 10 cm ultra low-attachment culture dish and cultured floating in RDM supplemented with 10% FBS. From D15 to D18, RDM was further supplemented with CHIR99021 (3 μM, Axon Medchem, Groningen, Netherlands) and SAG (100 nM, Enzo Life Sciences, Lörrach, Germany). At D18, cell aggregates were transferred to N2 medium (DMEM/F12 GlutaMAX, 1% N2, 1% pen/strep, 0.1% amphotericin B) and incubated at 40% O_2_. Retinal epithelial evaginations were manually isolated between D24 and D31 using surgical tweezers (Dumont No. 5) under microscope vision. From D24 onward, 10% FBS was added to the media. Synthetic retinoid analogue EC23 (0.3 μM) was added from D25 to D120. Media was changed every 2–3 days.

To assess the reliability of the CYST protocol, multiple independent experiments were performed with several hiPSC lines (Table 1). To compare the two organoid protocols (Supplementary Figures 4A, B), a parallel differentiation strategy was used: Cells from the same batch of undifferentiated hiPSCs were simultaneously used for HRO generation with both protocols [independent experiments (*N*) ≥ 2, hiPSC lines (L) = 4].

### SEM

Samples were fixed in 4% formaldehyde in 100 mM phosphate buffer, followed by post-fixation in modified Karnovsky (2% glutaraldehyde and 2% formaldehyde in 50 mM HEPES or 100 mM phosphate buffer), or fixed directly in modified Karnovsky fixative. For Scanning electron microscopy (SEM), samples were washed 2 × 5 min with PBS and 3 × 5 min with bi-distilled water, post-stained with 1% osmium tetroxide (OsO_4_) for 2 h on ice, washed several times in water and dehydrated in a graded series of ethanol/water mixtures up to pure ethanol (30, 50, 70, 96, and 3 × 100% on molecular sieve, 15 min each) and critical point drying using a Leica CPD300. Dried samples were mounted on 12 mm aluminum stubs using conductive carbon tabs, and additionally grounded with conductive liquid silver paint. To increase contrast and conductivity, samples were sputter coated with gold (BAL-TEC SCD 050 sputter coater, settings: 60–80 s, with 60 mA, at 5 cm working distance) or with platinum (coater settings: 40 s, with 40 mA, at 5 cm working distance). Finally, samples were imaged with a JSM 7500F scanning electron microscope (JEOL, Freising, Germany) running at 5kV (in-lens SE-detector, working distances between 3 and 8 mm).

### TEM

For transmission electron microscopy (TEM), samples were fixed as described above and further processed following a modified version of the Ellisman protocol for serial block-face SEM (Deerinck et al., 2010), generating enhanced contrasts by treatment with OsO_4_, thiocarbohydrazide (TCH), and again OsO_4_. In brief, samples fixed at least overnight in Karnovsky fixative were post-fixed in a 2% aqueous OsO_4_ solution containing 1.5% potassium ferrocyanide and 2 mM CaCl_2_ for 30 min on ice. After washes in water, samples were incubated in 1% TCH in water (20 min at RT), followed by washes in water and a second osmium contrasting step in 2% OsO_4_/water (30 min on ice). Samples were washed in water, contrasted en bloc with 1% uranyl acetate/water for 2 h on ice, washed again in water, dehydrated in a graded series of ethanol/water mixtures (30, 50, 70, 90, 96%), followed by three changes in pure ethanol on molecular sieve. Samples were infiltrated into epon 812 (epon/ethanol mixtures: 1:3, 1:1, 3:1 for 1 h each, followed by pure epon overnight, then pure epon for 5 h), embedded into flat embedding molds, and cured at 65°C overnight. Ultrathin sections (70 nm) were prepared with a Leica UC6 ultramicrotome (Leica Microsystems, Wetzlar, Germany), collected on formvar-coated slot grids, and stained with lead citrate and uranyl acetate. Contrasted ultrathin sections were analyzed on a Jeol JEM1400 Plus (JEOL, Germany, camera: Ruby, JEOL) running at 80kV acceleration voltage.

### Immunohistochemistry

Human retinal organoids were fixed in 4% PFA in PBS, cryoprotected in a graded series of sucrose solutions (10, 30, and 50% in PBS), embedded in OCT compound (Sakura Finetek, Umkirch, Germany), frozen at −80°C for 30 min and stored at −20°C. HROs were cryosectioned (12 μm thickness, Thermo Fisher, model NX70), mounted on Superfrost Ultra Plus slides (Thermo Scientific), and stored at −80°C. Sections were washed in PBS for 15 min and, if required, antigen retrieval was performed (10 mM sodium citrate, pH 6.0, 30 min at 70°C). Sections were incubated in blocking solution (0.5% BSA and 0.3% TritonX-100 in PBS, 1 h at RT; if required 0.01 mg/ml DNAse was added), followed by primary antibody solution (48 h at 4°C, Supplementary Table 2). After washing with PBS, sections were incubated with species-specific secondary antibodies (1:1000, 2 h at RT) produced in donkey (Dianova). Nuclei were counterstained with DAPI. Sections were washed in PBS, mounted with Fluoromount-G and coverslipped.

### Imaging

*Live imaging of epithelial cyst formation:* To monitor epithelial cyst formation, we used two transgenic reporter lines for actin (ACTB-mEGFP, Cat No. AICS-0016) and tight junction protein 1 (TJP1-mEGFP, Cat No. AICS-0023) developed at the Allen Institute for Cell Science (Roberts et al., 2017) and available through Coriell.^2^ Acutely dissociated hiPSC clusters were resuspended in Matrigel and seeded into a glass bottom dish (ibidi, Gräfelfing, Germany; μ-Dish 35 mm). The dishes were placed at 37°C for gelification in upright followed by inverted orientation for 5 min each before medium was added. Time-lapse imaging was performed using a Zeiss microscopy system based on an inverted Axio Observer Z1 with a Yokogawa CSU-X1 spinning disk confocal scanning unit and a cage incubator (37°C, 5% CO_2_). Imaging was done with a Plan Apo 20 × (0.8) objective with a frequency of one frame per hour. A 488 nm LED was used for fluorescence excitation.

*Live imaging microscopy of HROs:* Brightfield images of HROs live in culture were recorded with Olympus microscopes (models CKX41 and CKX53), 5 × objective and phase contrast. To study the development of potential inner and outer photoreceptor segments we recorded HRO wholemounts (5A line) in culture by differential interference contrast (DIC) imaging at an oblique angle (45° up to cross-sectional). Four HROs per time point were imaged using the live-imaging system described above. A DIC II condenser (NA = 0.55) was used, together with a Plan Apo 20 × (0.8) DIC II objective and a DIC II prism. Köhler illumination alignment was done prior to imaging. 3D reconstructions (projection) of optical stacks were performed using Fiji software.

### Quantitative analyses

Characterization and comparison between protocols and/or lines was performed by calculating several parameters, defined as follows:

*Mitosis*: Analysis was performed by counting phospho-histone-3 (PHH3+) cells in entire HRO sections (Supplementary Figure 6), and normalized to total DAPI + area (see below for binary image analysis) of the same section. Generally, for all data presented, we did not select organoids for analysis (i.e., we did not exclude those with extraretinal structures). Thus, entire HRO cross-section areas were used for analysis: The average DAPI + area of a D200 HRO section amounted to 0.157 ± 0.103 mm^2^ (*n* = 109).

*Total yield*: Total number of 3D structures (T3D) at the end time point divided by the number of starting hiPSC wells (6-well plate). To compare protocols, the end time point was either D200 (5A) or D90-D100 (CRTD1, CRTD2, CRTD3); for all other quantifications, the end time point was D200. *HRO yield*: Number of 3D structures harboring a retinal epithelium (HRO) at the end time point per starting hiPSC well. To distinguish retinal from non-retinal organoids, cryosections were immunostained with anti-RAX antibody, and scored either HRO (RAX + retinal epithelium) or non-retinal (RAX- 3D structures). Samples with few RAX + cells and with no distinctive epithelia were also scored as non-retinal.

*Fraction of RAX* + *retinal epithelium (%RE)*: To determine the percentage of retinal epithelium in each organoid at the end time point (see above), we measured the RAX + area in relation to total cell area as a proxy. Fluorescent images of RAX + and DAPI + nuclei were acquired with a 5 × Plan-Apochromat objective (Supplementary Figure 3B), Fiji software was used to create binary images by the default threshold algorithm (Supplementary Figure 5E) and to determine RAX + and DAPI + areas. Because RAX expression varies throughout retinal development (not shown), only age-matched HROs were used for this comparison. *Reactive gliosis*: Fluorescent images of HRO cryosections immunostained with anti-GFAP antibody were used to calculate the gliotic area (GFAP+) per total HRO area (DAPI +), in a similar manner to that to calculate the%RE value.

*Fraction of HROs with extra-retinal structures*: HRO cryosection images used to determine%RE, were also visually inspected and scored for the presence or absence of extra-retinal structures, which either showed no epithelial structure or a thin one- or two-cell nuclei layered structure that often corresponded to pigmented domains in the brightfield images (Supplementary Figure 5F).

*Quantification of defined cells of interest*: HRO sections were immunostained for CRX, ARR3, NRL, and SOX9; marker + cells were quantified in randomly chosen regions of interest (ROI) within the neuroepithelial domains. Each ROI consisted of a 100 × 200 μm (width × length) region, with the x-axis positioned parallel to the outer (apical) surface of the epithelium. ROI images are z-axis projections of 5 × 1 μm, i.e., five optical planes, 1 μm apart, and acquired in Apotome mode using a 20 × Plan-Apochromat objective (Zeiss Apotome2 microscope). For cell counts, maximum intensity projection of the optical planes was performed, and immunolabelled cells on the resulting images were manually counted using the cell-counter tool from Fiji software. If possible, two ROIs per HRO, positioned on opposing sides of the organoid, were imaged and quantified. The mean of two ROIs per individual organoid (n) was used for data plotting and statistical analysis. The number of independent experiments (N), HROs (n), and lines (L) analyzed per experiment are given in Supplementary Table 3.

*Photoreceptor inner (PIS) and outer (POS) segment analysis:* PISs and POSs were analyzed in ROIs using the cell-counter tool in Fiji software. In a first analysis approach immunostained HRO cross-sections were used. PISs were defined as individual RCVRN + photoreceptor protrusions that were located outside of the apical retinal epithelium border and did not co-localize with cell nuclei markers (DAPI-). The apical epithelial border was determined based on the MG marker RLBP1. In a second analysis approach, SEM images of D440 HRO wholemounts (5A line) were used that were acquired perpendicular to the organoid surface (en-face). Images with an oblique imaging angle were excluded. A grid was used to guide the SEM-based count, and PISs/POSs that were not entirely within the image area were counted only on two image borders. Potential cone PISs appeared larger and were more protruding than potential rod PISs. Additionally, rod PISs were partially hidden among the cone PISs, and thus likely underscored. Potential nascent POSs are identified as the smaller structures located on top of a PISs; only POSs clearly connected to the corresponding PISs were included. The total number of PISs was used to determine the PR/area ratio (mean ROI area: 47.000 μm^2^). To obtain the POS/PIS ratio, only intact PISs were considered, since a few of them (<1%) appeared to be broken (544–703 PISs were analyzed per ROI, 2 ROIs/n, *n* = 2 HROs). To obtain the average diameter of PISs and POSs, 50 of each were measured (1 ROI/n, *n* = 2 HROs).

### scRNA-seq and data analysis

*scRNA-seq:* HRO single-cell transcriptomics data preprocessing, visualization, and most downstream analysis were conducted using scanpy (version 1.3.1). Two HRO samples from the hiPSC line 5A (HRO1, HRO2; both D200) were used for the characterization and annotation procedure. The HRO1 sample has already been published (Völkner et al., 2022) and deposited on Gene Expression Omnibus under the sample name S3-CTRL_HRO_2 and accession code GSE174215. The HRO2 dataset was generated in the same way as HRO1, and scRNA-sequencing was performed together with HRO1. The raw expression data for HRO2 is deposited on Gene Expression Omnibus under the sample name S3_CTRL_HRO3 and accession code GSE237007. RNA sequencing reads were aligned to the human genome [human genome hg38 (Ensembl V87)] using the Cellranger software (v2.1.0). Source data are provided within this paper.

*Data preprocessing:* The data was processed using scanpy (version 1.3.1) following steps from the tutorial. Cells with fewer than 200 expressed genes and genes not expressed in at least three cells were discarded immediately. Based on the distribution of gene counts and the percentage of mitochondrial genes, cells with fewer than 2,500 genes and 4% mitochondrial genes were kept. The datasets were normalized, then highly variable genes were identified, log-transformed, and scaled.

*Cluster detection:* Principal component analysis (PCA) was used to reduce dimensionality. A neighborhood graph was calculated using ten neighbors and 40 principal components, and a UMAP embedding was computed. Cell clusters were detected using Louvain clustering (resolution parameter of 2).

*Cluster annotation:* Human retina organoids were annotated using a manual annotation and a transfer learning approach.

*Manual annotation.* Using literature and previous experiments, GOI lists of known marker genes were assembled. The overlap of shared genes between GOI lists and the single-cell data was used to analyze the expression pattern across clusters. To avoid differences in cell-cluster embedding and, therefore, re-evaluating the GOI expression patterns, scanpy (version 1.3.1) was used for preprocessing. Other visualizations were generated using scanpy version 1.4.1. Generally, the respective cell type was assigned if most cell type-specific marker genes revealed a unique expression in one Louvain cluster. Finally, all the Louvain clusters were manually annotated to one of the major populations: Cones; rods; Müller glia (MG); bipolar cells; amacrine, horizontal, retinal ganglion (AHG) cells, and the premature photoreceptor cluster.

*Transfer learning using CaSTLe*. Aiming to learn cell type-specific expression patterns from the data, we applied CaSTLe, a transfer learning approach (Lieberman et al., 2018). A fully-developed human retina organoid dataset (30- and 38-week-old HROs; Cowan et al., 2020) was used as a reference, including 37 different retinal sub-cell types across 44 000 cells. To increase the training performance, we reduced the resolution of retinal cell types (Supplementary Table 4). The annotation procedure was run following the provided GitHub tutorial. All model parameters were selected following the original publication using the multiclass implementation.

*Sample integration.* Following the annotation procedure, we integrated both HRO datasets. Here, the low-dimensional UMAP embedding of HRO1 was used as a basis. Both datasets were first filtered on the set of shared genes. Then a new low dimensional embedding was calculated based on the filtered HRO1 dataset. The ingest function from the scanpy tool was used to remove batch effects.

### Data analyses and presentation

Statistical analysis was performed with GraphPad Prism 7 software. To compare the means of three or more groups, a one-way ANOVA was used followed by Tukey’s *post hoc* multiple comparison test; to compare the means of three or more groups with two variables, a two-way ANOVA was used followed by Sidak’s *post hoc* multiple comparison test. To compare the means of two normally distributed groups, an unpaired two-tailed Student’s *t*-test was used. Supplementary Table 3 lists sample size, results [mean ± standard deviation (SD)], statistical data and test per figure. Results were considered significant for *p* < 0.05. Data graphs and schematic illustrations were prepared using GraphPad Prism 7 and Adobe Illustrator CS5 software, respectively. Images were optimized by making minor changes to contrast and brightness using Fiji or Adobe Photoshop CS5 software.

## Results

### Efficient and reproducible retinogenesis using the CYST protocol across multiple hiPSC lines

**FIGURE 1:**
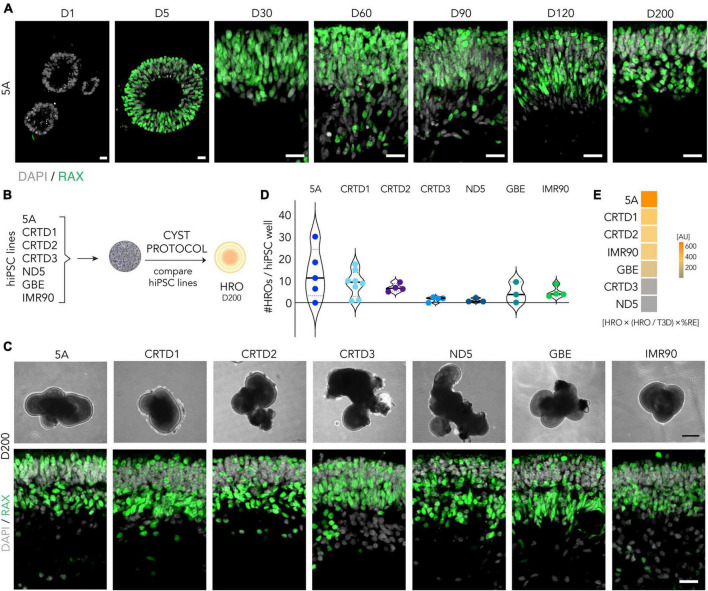
Efficiency of the CYST human retina organoid protocol across seven hiPSC lines. **(A)** RAX-immunostained cryosections of the 5A line at selected time points throughout retinal organoidogenesis: RAX- neuroepithelial cyst stage (D1), RAX + eyefield stage (D5), and early- to late-stage retinogenesis (D30-200). DAPI labels cell nuclei. **(B)** Scheme: Experimental paradigm for the data shown in panels **(C–E)**: To determine robustness of the CYST protocol, HRO differentiation from seven different hiPSC lines (5A, CRTD1, CRTD2, CRTD3, ND5, GBE, and IMR90) was compared (see Table 1). **(C)** Representative microscopic phase-contrast images of HROs in culture (upper panel); and images of RAX-immunostained HRO cryosections (lower panel). HROs were at D200 and derived from different hiPSC lines. **(D)** Violin plot: Total number of HROs generated per well of hiPSC (HRO yield) defined as the total number of 3D structures (T3D) with RAX + retinal epithelia (*N* = 3–8/hiPSC line). Related data in Supplementary Figure 3D. **(E)** The overall performance of all hiPSC lines in HRO generation was estimated by multiplying HRO yield, fraction of HRO yield per T3D, and%RE (defined as RAX + area per total cell area based on DAPI nuclear stain per HRO; Supplementary Figure 5E). Scale bars: **(A)**, 20 μm; **(C)**, upper panel, 200 μm, lower panel, 20 μm. AU, arbitrary units. Related data are provided in Supplementary Table 3.

A well-known but not yet completely understood source of variability in organoid and cell differentiation from pluripotent stem cells is differences between cell lines (Irion et al., 2008; Fossati et al., 2016; Hernandez et al., 2022): This has been reported for several retina organoid systems (Meyer et al., 2011; Hallam et al., 2018; Capowski et al., 2019; Mellough et al., 2019; Cowan et al., 2020). For example, retinal optic cup formation with neural retina and opposing retinal pigment epithelial cells has so far only been reproducibly achieved with one mouse pluripotent stem cell line (Eiraku et al., 2011), and does not occur in others (Hiler et al., 2015; Völkner et al., 2016). Further, a highly efficient HRO system dominated by rod photoreceptors appears to work best with four out of 23 hiPSC lines tested (Cowan et al., 2020). Here, we sought to further characterize HRO generation by our previously established system (Völkner et al., 2022) based on neuroepithelial cysts (CYST protocol) by comparing different hiPSC lines. The CYST protocol starts by acute dissociation of adherent cultures of undifferentiated hiPSCs into small cell clusters which, upon resuspension in Matrigel, spontaneously form neuroepithelial cysts with a single central lumen within the first 3 days. To confirm formation of a neuroepithelial eyefield and retina in histological studies, we used the pan-retinal transcriptional factor RAX [retina and anterior neural fold homeobox (Bailey et al., 2004; Figure 1A)]. RAX is expressed during the early development of the eyefield, throughout retinogenesis, and in the mature retina (Nakano et al., 2012). Conversely, 3D structures without RAX or with only a few RAX + cells scattered throughout the post-mitotic organoid were scored non- or extraretinal. To demonstrate epithelial cyst formation, we recorded movies using brightfield and fluorescence live-imaging microscopy. A transgenic reporter hiPSC line expressing green fluorescent protein (GFP)-tagged beta actin (ACTB) facilitates monitoring of all cells: Upon cell dissociation, clusters of 2D hiPSCs rapidly form spherical 3D epithelial structures within hours which develop an intraepithelial lumen within a day (Supplementary Figure 2A; Supplementary Movie 1). hiPSCs expressing GFP-tagged tight junction protein 1 (TJP1) show accumulation of GFP at the intraluminal cell border starting at about 12–24 h supporting formation of a polarized epithelial cyst (Supplementary Figure 2B; Supplementary Movie 2). Notably, all hiPSC lines used in this study were able to form neuroepithelia and differentiate into RAX + retina when exposed to the CYST protocol (Figures 1B, C; Supplementary Figures 2A, B).

To quantitatively assess hiPSC-line dependence, we scored retinal differentiation during the culture of seven hiPSC lines at day (D) 200 (*N* ≥ 3 per hiPSC line, Figure 1B; Supplementary Table 3): 5A, CRTD1, CRTD2, CRTD3, ND5, GBE, and IMR90, which differ in the cell of origin and the reprogramming method (Table 1). In culture, HROs could be identified by their bright epithelial structure (Figure 1C). Their size increased, and their morphology changed throughout development and culture time comparable to previous reports (Capowski et al., 2019). However, differences between HROs from different hiPSC lines at any given developmental age could be observed live in culture (Figure 1C; Supplementary Figure 3A) which were consistent between experiments. These differences involved the brightness and thickness of the epithelium, as well as the infrequent presence of extra-retinal structures. While four hiPSC lines showed rather homogenous retinal epithelia at D200, HROs from the CRTD2, CRTD3, and more strongly ND5 lines frequently showed more complex and convoluted morphologies with multiple retinal, non-retinal, and extra-retinal domains. Notably, these differences were not visible at an early stage in all cases: Between D30 and D90, epithelia derived by the ND5 line appeared rather inhomogeneous, but all others looked quite comparable (Supplementary Figure 3A). Independently of the hiPSC line, HROs at D200 of differentiation showed RAX + cells organized into discernable outer and inner nuclear layers across organoid epithelia, which are characteristic for the vertebrate retina (Bringmann et al., 2018). RAX was consistently most highly expressed in the inner nuclear layer (INL) and in the most apical cells of the outer nuclear layer (ONL) (Figure 1C; Supplementary Figures 3B, C).

To quantify differences between hiPSC lines, we compared the HRO yield achieved by each line at D200. HRO yield was defined as the total number of 3D cellular structures (T3D) with RAX + retinal epithelia assessed by immunostaining (Figure 1D; *N* ≥ 3 experiments per cell line) that are produced per starting cell culture well of hiPSCs (one well of a 6-well culture plate). Notably, all hiPSC lines produced HROs, which ranged between 13 and 7 (5A > CRTD1 > CRTD2) or 5 and 1 (IMR90 > GBE > CRTD3 > ND5) HROs per hiPSC well. For example, the 5A line yielded most HROs per round of differentiation: On average 13 ± 12 (mean ± SD; *N* = 5) HROs; with a maximum of 30 and minimum of zero. To depict the variability, we calculated the coefficient of variation (COV; ratio of SD to mean) for the HRO yield across all lines (70%) and for each hiPSC line: 5A 88%, CRTD1 67%, CRTD2 27%, CRTD3 69%, ND5 85%, GBE 106%, and IMR90 52%. To determine the proportion of neural retina within each HRO, we measured the RAX + area per total cell area, based on DAPI nuclear stain (%RE) as a proxy: HROs from six hiPSC lines showed similar levels of retinal proportion (5A 62 ± 15%; CRTD1 59 ± 20%; CRTD2 63 ± 18%; CRTD3 54 ± 18%; GBE 57 ± 14%; IMR90 66 ± 15%); only the ND5 line (43 ± 20%) was slightly lower than the others (Supplementary Figure 3D; Supplementary Table 3). Further, we assessed how many non-retinal organoids, defined as RAX- 3D cellular structures, are generated in addition to HROs: Some lines produced 97–100% HROs in all independent experiments (i.e., 5A, GBE, and IMR90) and others showed lower efficiency, 74–83% HROs on average (e.g., CRTD2 and ND5, Supplementary Figure 3D). To summarize all the above data on cell line-dependent HRO quality and quantity, we calculated an overall retinal differentiation score for each hiPSC line by multiplying HRO yield, fraction of HRO yield per T3D, and%RE (Figure 1E). This score shows that five lines (5A, IMR90, GBE, CRTD1, CRTD2) are better performers than the others (CRTD3, ND5). Based on our data, we could not detect any relationship between the efficiency of HRO production of a given hiPSC line and its cell type of origin, derivation method, or donor gender (Table 1), but more detailed analyses might be able to determine this. In conclusion, comparing HROs from different hiPSC lines demonstrated that all seven hiPSC lines tested reproducibly generate HROs using the CYST protocol, but some lines are more efficient than others.

### Exploring HRO protocol efficiency through parallel differentiation: CYST vs. AGG protocol

**FIGURE 2:**
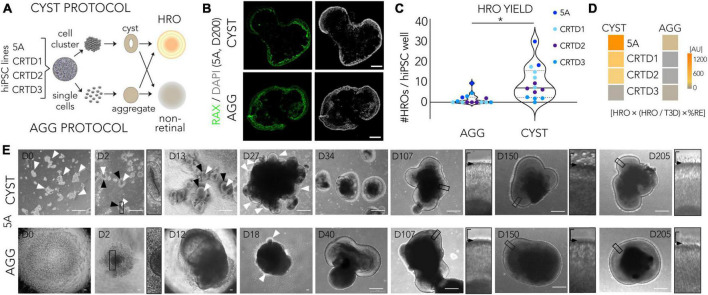
Assessment of retina organoid protocol performance using side-by-side comparison. **(A)** Experimental paradigm for the parallel differentiation approach: To quantitatively assess the efficiency of human retina organoid (HRO) production, hiPSCs from the same batch were differentiated into HROs using the two different protocols in parallel. We started to explore HROs derived from hiPSC cluster-derived neuroepithelial cysts (CYST protocol) compared to HROs produced from single cell-derived aggregates (AGG protocol). **(B)** Representative images of HRO cross-sections from the two protocols immunostained for the retinal marker RAX; nuclei were labeled with DAPI (images derived from 5A line). **(C)** Comparison of the HRO yield derived per parallel differentiation between the two protocols. HRO yield was assessed on immunostained sections and defined as the total number of 3D cellular structures (T3D) consisting of RAX + retinal epithelia (*N* = 2–4/hiPSC line). The following hiPSC lines were used: 5A, CRTD1, CRTD2, CRTD3 (Table 1). Statistical analysis results are given as follows: *p < 0.05 (Student’s *t*-test). **(D)** The overall performance of all hiPSC lines in generating HROs was estimated by multiplying HRO yield, fraction of HRO yield per T3D, and%RE (defined as RAX + area per total cell area based on DAPI nuclear stain per HRO; Supplementary Figure 5E). **(E)** Representative brightfield images of the two protocols; representative images shown are based on the 5A line. CYST protocol: hiPSC colonies are dissociated into multiple cell clusters [day (D) 0, arrowheads] which spontaneously form neuroepithelial cysts [D2 and D13; bright neuroepithelia (black arrowheads) with a single lumen (white arrowheads)] upon Matrigel embedding. At D2, a selected region of interest is provided at higher magnification (white arrowheads and black-boxed area) to highlight epithelial cysts and aggregate formation in the CYST and AGG protocols, respectively. At D5, cysts are plated for attached culture until D13. Around D30, expanded HRO epithelia in free-floating culture (arrowheads; seen at D27 but not yet at D18) are manually isolated, further expanding in free-floating culture (D34, D107, and D205). AGG protocol (5A line): hiPSC colonies are dissociated into single cells (D0), which are aggregated (D2) and aggregates subsequently further expand in size (D12). Emerging HRO epithelia (D18, arrowheads) are manually isolated and cultured free floating (D40, D107, and D205). Higher-magnification images of randomly-selected ROIs (black-boxed area in main image) are presented to show the apical HRO boundary (black arrow) and monitor photoreceptor inner segment development above it. AU, arbitrary units. Scale bars: **(B)**, 500 μm; **(C)**, 200 μm. Related data are provided in Supplementary Table 3.

To assess the efficiency of the CYST protocol from a different perspective, we started to contrast it with another, conceptually different HRO protocol. There are several reasons, outlined above, for an unbiased systematic quantitative comparison (Figure 2A; Supplementary Figures 4A, B): The CYST protocol is based on generating hiPS cell cluster-derived neuroepithelial cysts (Zhu et al., 2013; Lowe et al., 2016; Völkner et al., 2021b,^2022^; Wagner et al., 2022). In contrast, one of the pioneering HRO protocols starts from aggregates of a defined number of acutely dissociated, single pluripotent stem cells (Nakano et al., 2012) and many others start from cell clumps derived by dissociation of pluripotent stem cells (Meyer et al., 2011; Zhong et al., 2014; Reichman et al., 2017). Here, we used the protocol by Nakano et al. (2012), herein termed the AGG (aggregate) protocol, for this comparison: This protocol was already established in our lab (Völkner et al., 2016). To avoid any potential effects related to hiPSC passage, we performed parallel differentiations by subjecting the same batch of hiPSCs to the CYST and AGG protocols simultaneously. To start exploring this protocol comparison strategy, here we selected and tested four hiPSC lines based on our data above: Using the CYST protocol, 5A and CRTD1 were the most efficient at producing HROs, although variable (Figure 1D); whereas CRTD2 and CRTD3 showed an average to low efficiency. Here, we quantified the same parameters at selected time points, as established above for cell line comparison (Figure 1; Supplementary Figures 3A, B, D). Most notably, the CYST protocol reproducibly resulted in a higher yield of HROs per hiPSC well across all four hiPSC lines tested, in comparison to the AGG protocol (mean ± SD: CYST, 10 ± 8; AGG, 2 ± 3; *N* = 14; *p* < 0.002; Figures 2B, C) using RAX immunostaining to identify the number of HROs among all 3D cell structures generated (Supplementary Table 3; Supplementary Figures 5A–C). Further, for most of the hiPSC lines used the total number of 3D cellular structures (T3D) was significantly higher in the CYST than the AGG protocol (Supplementary Figure 5A; CRTD1, *p* = 0.001; CRTD2, *p* = 0.009; CRTD3, p>0.99, 5A, *p* = 0.04; Supplementary Table 3). There was no difference in the percentage of retinal epithelium per 3D cellular structure (%RE) among individual HROs within each protocol (Supplementary Figures 5D, E). Extra-retinal structures, here defined as non-epithelial or one- or two-cell layered pigmented domains, were observed in both protocols depending on the hiPSC line (Supplementary Figure 5F). These structures might be retinal or ciliary pigment epithelial cells, as previously observed (Nakano et al., 2012; Kuwahara et al., 2015; Lowe et al., 2016). In summary, we calculated an overall retinal differentiation score for each hiPSC line by multiplying HRO yield, fraction of HRO yield per T3D, and%RE: Based on these parameters, three out of four lines analyzed here performed better when subjected to the CYST compared to the AGG protocol (Figure 2D).

**FIGURE 3:**
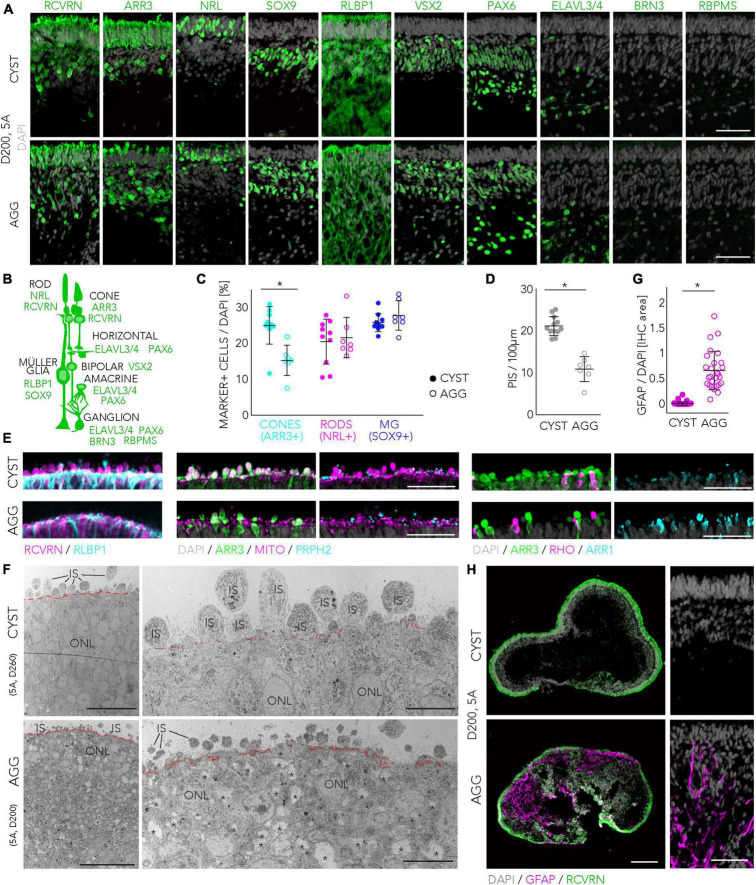
Retinal cell composition and structure of organoids derived from the CYST and AGG protocols. **(A–C)** Major retinal cell types were observed in HROs derived from the 5A hiPSC line at D200 in both protocols: Cone (ARR3+) and rod (NRL+) photoreceptors (RCVRN+); Müller glia (MG; SOX9 + and RLBP1+); bipolar (VSX2+), amacrine and horizontal cells (PAX6 + and ELAVL3/4+). Retinal ganglion cells (RBPMS + , BRN3 + , PAX6 + , and ELAVL3/4 +) were absent at D200 but were found at earlier stages (Supplementary Figure 5G). Representative images show selected regions of interest (ROIs) of HRO sections **(A)**. Schematic drawing of retinal cells and markers used for their identification **(B)**. Frequently expressed markers are shown for the major cell types; however, some markers are occasionally also expressed by others: Bipolars (RCVRN), and MG (PAX6, VSX2) cells. Quantitative analysis of cone, rod and MG cells in ROIs of immunostained HROs derived by the CYST and AGG protocols **(C)** [representative images shown in panel **(A)**] (NRL and ARR3 were stained and analyzed together; *n* = 7–10 HROs/N, *N* = 2). **(D,E)**: Data plot **(D)** and representative images **(E)** for the analysis of photoreceptor inner (PIS) and outer (POS) segments. PISs were quantified based on RCVRN + protrusions above the most apical retinal epithelial region (depicted by immunostaining for the Müller glia marker RLBP1) in HRO sections. RCVRN labels all photoreceptors (rods and cones). ARR3 + protrusions colabeled with staining for mitochondria and PRPH2 indicate cone PISs and POSs. ARR1 + and/or RHO + protrusions indicate rod PISs and POSs (*n* = 7–12 HROs/N, *N* = 2). **(F)** Transmission electron microscopy (TEM) images of HRO sections from CYST (at D260) and AGG (at D200) protocols derived from the 5A hiPSC line, depicting epithelial structure and cell connections at the apical HRO border, forming an outer limiting membrane (higher magnification, red lines), above which inner segments (ISs) were observed. Retinal structural irregularities observed in AGG-derived HROs are indicated by asterisks. **(G,H)** Assessment of reactive gliosis by GFAP immunostaining (magenta) in HROs derived from both protocols at D200; representative images (HRO cryosection overview and ROI) and quantification (*n* = 27–46/N, *N* = 4). Scale bars: **(A,E)**, 50 μm; **(F)**, overviews, 50 μm, magnification, 10 μm; **(H)**, overviews, 200 μm, ROIs, 50 μm. IHC, immunohistochemistry; IS, inner segment; ONL, outer nuclear layer. All representative images and quantifications were done using the 5A line. Statistical analysis results (Student’s *t*-test) are given as follows: *p < 0.0001. Related data are provided in Supplementary Table 3.

We then studied some selected parameters of HROs generated by both protocols and derived from the 5A line in more detail: Visual inspection of HROs revealed morphological differences (Figure 2E; representative images 5A line), not only at early stages, where either cysts or aggregates are generated (D0 and D2), but also throughout HRO development: CYST-derived HROs tended to be smaller at any given time point and the retinal neuroepithelium tended to be brighter in CYST-derived HROs (Figure 2E, D34/40). At later stages, CYST-derived HROs showed a more pronounced separation into an outer and inner retinal nuclear layer (Figure 2E, D205, higher magnification ROIs). As previously reported (Capowski et al., 2019), we also observed an intermediate phase of general darkening of living neuroepithelia by phase contrast microscopy (Figure 2E, D107). Notably, we observed a difference in the development of the cell protrusions, potentially the outer and inner photoreceptor segments (explained in more detail below; Wahlin et al., 2017; Capowski et al., 2019), emerging from the apical edge (the outer limiting membrane, indicated by arrowheads in Figure 2E) of the HROs starting at around D150: This was more pronounced at advanced culture stages in the CYST but not AGG protocol (Figure 2E, D205, higher magnification ROIs). Next, we determined if HROs derived from the 5A line using both protocols contain all of the major retinal cell types, properly organized into three nuclear layers (Figures 3A, B; *N* = 4): Immunostaining [marker positive (+)] analysis of HROs at D200, showed that cone (ARR3+) and rod (NRL+) photoreceptors (RCVRN+) were localized in the ONL, Müller glia (SOX9 + and RLBP1+), bipolar (VSX2+), amacrine and horizontal cells (PAX6 + and ELAVL3/4 +) were present in the INL. Although retinal ganglion cells (BRN3 + , RBPMS + , PAX6 + and ELAVL3/4 +) are generated during HRO development and thus frequently observed at around D90 (Supplementary Figure 5G), they were almost absent in the innermost layer of D200 HROs. Quantitative comparison of selected cell-type markers normalized to total cell count (based on cell nuclei labeled by DAPI; *n* ≥ 7 HROs; *N* = 2) of the CYST versus AGG protocol at D200 (Figure 3C) show a 1.7-fold higher number of cones (ARR3 + ; mean ± SD: 25 ± 5% vs. 15 ± 4%; *p* < 0.006), and comparable number of rods (NRL + ; 21 ± 6% vs. 22 ± 6%) and Müller glia (SOX9 + ; 26 ± 2% vs. 28 ± 4%). Notably, this parallel comparison confirms the original results from the AGG protocol (Nakano et al., 2012) and our data on the CYST protocol (Völkner et al., 2022; Supplementary Table 3): AGG generates a rod-dominant HRO system, CYST generates one richer in cones. Further, RCVRN staining confirmed PIS formation at D200 in both protocols (Figures 3D, E), although the overall quality of PIS morphology and their number appeared higher in HROs from the CYST protocol. Quantitative analysis of PISs supports this (Figure 3D; CYST: mean ± SD 21 ± 2; AGG: 11 ± 3; *p* < 0.0001, *n* ≥ 7 HROs; *N* = 2). Further markers supported potential differential POS/PIS formation: PRPH2, which is a main structural component of the POSs, was found in proximity to mitochondria-rich PISs (Figure 3E). Evaluation of staining for ARR1 and RHO as well as ARR3 indicates that rods and cones, respectively, form PISs (Figure 3E). Ultrastructural analysis by TEM suggest a high degree of nuclear organization within the ONL of CYST-derived HROs; this is also indicated by ordered cellular connections between apical Müller glia cell processes and photoreceptor cells (Figure 3F, red dashed line). Photoreceptor cell structures appeared comparable to the vertebrate retina *in vivo* (Hoshino et al., 2017; Bringmann et al., 2018; Cowan et al., 2020): Photoreceptor cell somata are localized in the ONL and PISs are above the OLM. PISs seemed more homogenous in size and morphology in CYST-derived HROs (Figure 3F), further supporting our immunostaining data (Figures 3A, E). Notably, a preliminary study of TEM images suggests tissue structural irregularities and gaps in the ONL of AGG- but not CYST-derived HROs (Figure 3F, asterisks), which might indicate some neuronal cell impairment. To further explore protocol-induced differences in the quality of the retinal structure, we analyzed the expression of glial fibrillary acidic protein (GFAP), which becomes upregulated upon neuronal pathology by Müller glia cells, and is a well-known hallmark marker of reactive gliosis (Bringmann et al., 2009; Karl and Reh, 2010). Gliosis is an umbrella term for various beneficial and detrimental glial functions. Importantly, no GFAP was detected in immunostained CYST-derived HROs at D200 in this work (Figures 3G, H) and up to D260 previously (Völkner et al., 2022), whereas GFAP was significantly upregulated in AGG-derived HROs at D200 (Figures 3G, H; *p* < 0.0001, *N* = 4, *n* ≥ 27/N; Supplementary Table 3). Of note, the original publication of the AGG protocol (Nakano et al., 2012) showed overall normal histological organization up to D126, but the presence of Müller glia cells and gliosis was not yet investigated. In our hands, however, we observed differences in overall retinal structure and reactive gliosis in HROs at D200 from the AGG protocol suggesting the development of spontaneous (potentially pathologic) changes but the reason for this is not yet known. More detailed studies are necessary to validate our results, and to determine the onset and frequency the observed potential pathologic changes, for example, if these already occur at D126. Generally, our data raise the question if maintenance of HROs in culture might become limited at some point by spontaneous pathology, and if so, whether HRO longer-term stability might differ between protocols, as previously observed in mouse retina organoid systems (Ito et al., 2017; Völkner et al., 2021a).

In conclusion, identifying key quantifiable parameters and comparing retinal differentiation from different hiPSC lines in parallel is a potential strategy for an unbiased assessment of the efficiency of an HRO protocol. We demonstrate a strategy for parallel comparison of different HRO protocols that revealed potential differences in production efficiency, overall quality, and maintenance. However, a larger scale and more detailed protocol comparison is necessary to validate our results. For example, for a representative comparative protocol study many more hiPSC lines need to be thoroughly tested in multiple rounds of differentiation and at best independently by multiple laboratories.

### Stem-cell proliferation and retinal generation dynamics in the CYST-based organoid system

**FIGURE 4:**
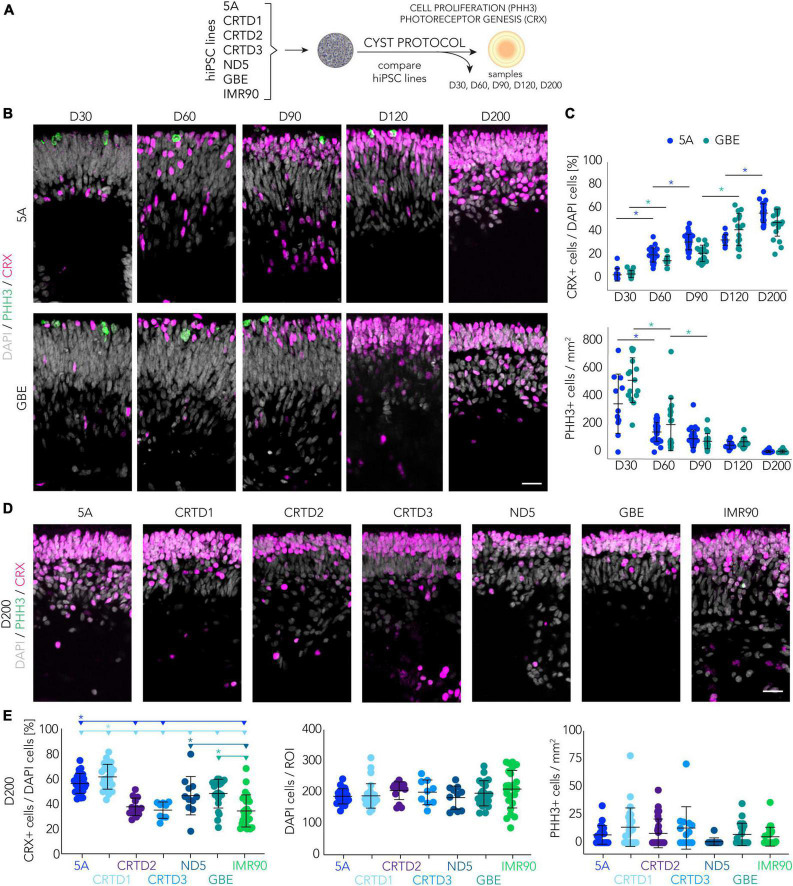
Retinogenesis dynamics in the CYST-based human retina organoid system. **(A)** Schematic of the experimental paradigm to assess the spatiotemporal development of HROs in the CYST protocol as shown in panels **(B–E)**: Progenitor cell proliferation and photoreceptor generation were investigated in seven hiPSC lines as denoted and at selected days **(D)** of development. **(B–E)** Photoreceptor genesis was studied by immunostaining for CRX, expressed in mature photoreceptors and their precursors. CRX + cells were counted per region of interest (ROI) in HRO cryosections and normalized to the number of DAPI + cells. Each dot in the plots represents the average of two ROIs per HRO (n) analyzed per experiment (N). Cell proliferation was assessed on HRO sections by counting cells immunopositive for the mitosis marker phospho-histone H3 (PHH3), normalized to total DAPI area of the same section. **(B,D)** Representative microscopic images (ROIs) of HRO cryosections derived from the hiPSC lines indicated and immunostained for PHH3 and CRX; related to the quantifications shown in panels **(C,E)**. **(C)** Quantitative analysis across retinogenesis. Asterisks (*) indicate a statistically significant (*p* < 0.05) difference for the color-matched hiPSC line between the two subsequent time points (N per time point: 5A, *N* = 1–3; GBE *N* = 2–3; 1-way ANOVA, Tukey *post-hoc* test; see Supplementary Table 3). **(E)** Quantitative analysis of D200 HROs. The horizontal lines and asterisks (*) above the scatter plots indicate a statistically significant (*p* < 0.05) difference between a selected (color-matched) hiPSC line and other hiPSC lines (*N* = 1–4 for each hiPSC line; 2-way ANOVA, Tukey *post-hoc* test; details in Supplementary Table 3). **(C,E)** Not all statistic data are shown; complete related data are provided in Supplementary Table 3. Note that the CRX and PHH3 datasets for D200 for lines 5A and GBE are the same in the plots in panels **(C,E)**. Scale bars: **(B,D)**, 20 μm. Related data are provided in Supplementary Table 3.

To gain an insight into the robustness and variance of CYST-generated HROs, we assessed the kinetics of organoid development (Figure 4A). We first sought to determine suitable time points to monitor progress and completion of retinal organoidogenesis, and then used these to compare several hiPSC lines. To achieve this, we investigated progenitor cell proliferation and photoreceptor cell genesis based on the mitotic marker phospho-histone-3 (PHH3) and the pan-photoreceptor marker cone-rod homeobox (CRX) transcription factor, respectively. Mitosis is initially high in *in vivo* retinal development, declines over time, and becomes absent upon stem-cell depletion (Hoshino et al., 2017; Sridhar et al., 2020). Accordingly, CRX becomes expressed in increasing numbers of proliferating progenitor cells, mostly committed to the photoreceptor but also bipolar fate; its expression is maintained in post-mitotic cones and rods (Chen et al., 1997; Glubrecht et al., 2009; Gagliardi et al., 2018; Yamamoto et al., 2020). We use two randomly-selected hiPSC lines (5A and GBE) to gain insight across development: We counted PHH3 + and CRX + cells on immunostained HRO sections sampled at multiple time points throughout development (Figures 4B, C). The percentage of CRX + cells over total (DAPI+) cells in the HROs increased throughout retinogenesis: At D30, 4 ± 4%; D60, 19 ± 6%; D90, 28 ± 8%; D120, 38 ± 12%; and D200, 52 ± 11% (mean ± SD for 5A and GBE hiPSC lines; Figure 4C). Concurrently, the number of PHH3 + cells per HRO section showed a significant reduction, indicative of retinal progenitor depletion over time (e.g., D30, 447 ± 201 cells/mm^2^ compared with D200, 7 ± 9; mean ± SD from 5A and GBE lines) (Figures 4B, C). In conclusion, the first CRX + photoreceptor cells are generated after about D30, following a period of progenitor cell expansion: Numbers increase from D60 until D200. Based on these data, we conclude that cell generation in HROs is completed before D200, but after D120, and thus we hypothesize that the analysis of both time points might be adequate to identify potential differences between different hiPSC lines in the dynamics (D120) and completion (D200) of retinogenesis.

To test this, we quantified PHH3+, CRX+, and total (DAPI) cells across different hiPSC lines (Figures 4D, E; Supplementary Figure 6A). Supporting our hypothesis: HROs from seven different hiPSC lines showed depletion of mitotic cells at D200, indicating that most of the cells were post-mitotic at this time point. Further, the percentage of CRX + cells in HROs at D200 from lines CRTD1 (62%) and 5A (56%) was slightly higher than in lines ND5 (46%) and GBE (48%). In comparison, the number of CRX + cells in CRTD2 (37%), CRTD3 (35%), and IMR90 (34%) were lower (Figure 4E). On average, these values are almost twice those found at comparable time points in previous studies with other HRO protocols (Supplementary Table 1), although one other protocol produced even higher amounts of CRX + cells (Phillips et al., 2018; Supplementary Table 1). The reason for such differences is not yet known: It could be due to protocols and/or hiPSC lines. Further, analysis of HROs derived from five selected different hiPSC lines at D120 still found many mitotic cells (Supplementary Figures 6B, C). The number of these cells varied considerably between hiPSC lines (from 40 per mm^2^ area of an HRO section in ND5 to 110 in IMR90). Further, three of the lines (5A, CRTD1, and ND5) had a higher percentage of CRX + cells at D200 than at D120 (Figure 4E; Supplementary Figure 6E; Supplementary Table 3), supporting the hypothesis that photoreceptor and bipolar production continues after D120 in HROs from some hiPSC lines. In summary, we observed a progressive decrease in the pool of mitotic cells and a concomitant increase in the number of CRX + cells in developing HROs from different hiPSC lines, indicating a dynamic retinogenesis with progenitor depletion, increasing neurogenesis, and reproducible completion (post-mitosis) at D200.

### Reliability of cellular composition of retina organoids generated by the CYST protocol

We sought to determine if CYST-derived HROs from different hiPSC lines and rounds of differentiation show a comparable, different, or variable retinal cell composition after the end of retinogenesis at D200. For example, retinal cell composition might vary due to hiPSC genotype or due to morphological changes as observed during organoidogenesis in some hiPSC lines (Figure 1C; Supplementary Figure 3A).

**FIGURE 5:**
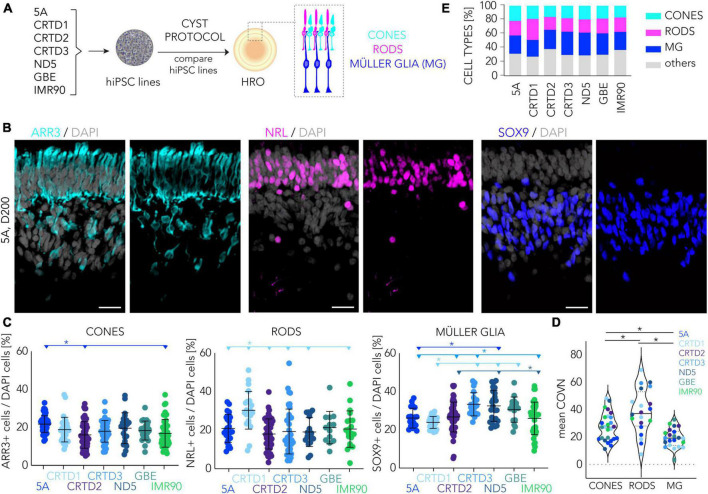
Cell composition analyses of human retina organoids across multiple hiPSC lines. **(A)** Schematic of the experimental paradigm to compare retinal composition in D200 HROs derived from seven different hiPSC lines. **(B)** Representative images depicting region of interests (ROIs) of HRO sections (derived from the 5A hiPSC line) immunostained for cone (ARR3), rod (NRL), and Müller glia (SOX9) markers. **(C)** Scatter plots showing quantitative analysis based on images shown in panel **(B)**. Each dot represents the average of two ROIs for a given HRO analyzed per experiment (*n* = 15–50 HROs/N, *N* = 2–7 experiments per line). **(D)** Coefficient of variation (COVN) calculated for each experiment (N) analyzed in panel **(C)**. **(E)** Graphical summary based on data shown in panel **(C)**: Most lines have about 20% cones, 20% rods, 30% Müller glia, and 30% other cells. **(E)**: Percentage of cones, rods and Müller glia (MG) cells across seven different hiPSC lines. Summary of the data presented in panel **(C)**. Statistical analyses (1-way ANOVA, Tukey *post-hoc* test) results **(C,D)** are given as follows: The horizontal lines and asterisks (*) above the scatter plots indicate a statistically significant (*p* < 0.05) difference between the selected (color-matched) hiPSC line and other hiPSC lines. HRO, human retina organoid; MG, Müller glia. Scale bar: **(B)**, 20 μm. Related data are provided in Supplementary Table 3.

Since the human retina shows considerable regional differences in photoreceptor cell distribution *in vivo*, we studied this quantitatively across HROs derived from the seven hiPSC lines. In humans, cone density is highest in the rod-free foveola, rod density increases with increasing eccentricity and peaks 3–5 mm away from the foveola, and the density of both photoreceptor types declines toward the periphery (Curcio et al., 1990; Jonas et al., 1992). To quantitatively assess HRO cell composition, we counted the number of ARR3 + cones (Sakuma et al., 1996), NRL + rods (Mears et al., 2001), and SOX9 + Müller glia cells (Poche et al., 2008; Völkner et al., 2022) in two randomly-selected ROIs on several immunostained HRO sections from seven hiPSC lines (Figure 5; Table 1). The total number of photoreceptor cells (ARR3 + and NRL+) was consistent across all hiPSC lines (mean ± SD 40 ± 13%; range 17–88%; *N* = 24, *n* = 189). Histological analysis also showed a correct localization of cones and rods in the ONL, and Müller glia in the INL (Figure 5B). On average, we detected that the percentage of cones per total cells (ARR3 + /DAPI + cell nuclei; mean ± SD) ranged between 22 ± 5% in HROs from the 5A line to 16 ± 7% in the CRTD2 line (Figure 5C; Supplementary Figure 7). Rod cell numbers were highest in the CRTD1 line (NRL + ; mean ± SD 30 ± 10%) and lowest in the CRTD2 line (18 ± 8%; Figure 5C). HROs derived from the CRTD3, ND5 or GBE lines had slightly more Müller glia cells (SOX9 + ; mean ± SD: CRTD3 33 ± 6%; ND5 33 ± 8%; GBE 31 ± 7%) than the others (CRTD1 24 ± 3%; 5A 26 ± 5%; IMR90 26 ± 9%; CRTD2 27 ± 7%; Figure 5C). To gain some insight into the variability, we calculated the coefficient of variation (COV; ratio of SD to mean in%) for each cell-type marker between experiments (Figure 5D; Supplementary Figure 7): Across all HRO differentiations (*N* ≥ 24) in the seven hiPSC lines, cones, rods, and Müller glia showed mean COVs of 28%, 41%, and 19%, respectively). For comparison, the top performer line 5A (Figure 1E), showed COVs of 20%, 26%, and 20% for cones, rods, and Müller glia, respectively (Supplementary Figure 7). Notably, Müller glia are the least variable cell type at all levels: Interorganoid (23%, mean of all HROs, *n* = 170), hiPSC line (19%, mean of all COVs per hiPSC line, *L* = 7), and experiments (19%, mean of all COVs per experiment, *N* = 20), whereas rods are twice as variable (48, 41, and 39%, respectively). Taking the average of all three cell markers in HRO at D200, the 5A (22%), GBE (23%) and CRTD1 (24%) lines were less variable between experiments than ND5 (31%), IMR90 (35%), CRTD3 (36%) and CRTD2 (33%). Interestingly, although HRO yield is quite variable between HROs from different hiPSC lines (Figure 1D), the variance in HRO cell composition (Supplementary Figure 7) does not seem to be related to this.

Next, we sought to determine if all the other major retinal cell types are present and if so, are they organized into three discernable nuclear layers in HROs from the seven hiPSC lines at D200. Thus, we qualitatively assessed if immunostained HROs are positive (+) for selected markers (Supplementary Figures 8, 9): RLBP1 staining shows that Müller glia span the entire retinal epithelial width; bipolar cells (VSX2+) were localized in the INL. Further, we observed potential amacrine, horizontal, and retinal ganglion cells (RGC) (PAX6 + and ELAVL3/4+) in the INL and innermost layer. More specific markers for RGCs (RBPMS + , BRN3+) showed that these were almost absent in D200 HROs from all hiPSC lines: if so, they were infrequently localized in scattered regions in the innermost layer (Supplementary Figure 8). Analysis of selected 5A-derived HROs at earlier and later time points throughout organoidogenesis (D30, D100, D120, D160, D210, D260) indicated that amacrines (ELAVL3/4) and RGCs (RBPMS, BRN3) are generated but there are almost none RGCs left by the end of retinogenesis (Supplementary Figure 9). In summary, HROs at D200 across HROs from all seven hiPSC lines harbored on average (mean ± SD, N>23) 18 ± 2% cones, 21 ± 4% rods, 29 ± 4% Müller glia (a ratio of 1:1.2:1.6) at D200: 32 ± 4% were therefore other cells (Figure 5E). For comparison, cones represent on average 5–6% of all photoreceptors in the human retina (Curcio et al., 1990). Further, the cone to rod ratio is about 1:20 in the peripheral regions. In contrast, it ranges from about 1:1 to 1:8 across the macular region, and the very center of the macula (the foveola) is composed solely of cones (for review see Supplementary Figures 3I–K in Völkner et al., 2022). Thus, our data presented here further confirm our previous observation (Völkner et al., 2022) that the CYST protocol provides HROs richer in cones across several hiPSC lines and independent differentiations (between a 1:1 and 0.6:1 cone-to-rod ratio depending on the cell line).

**FIGURE 6:**
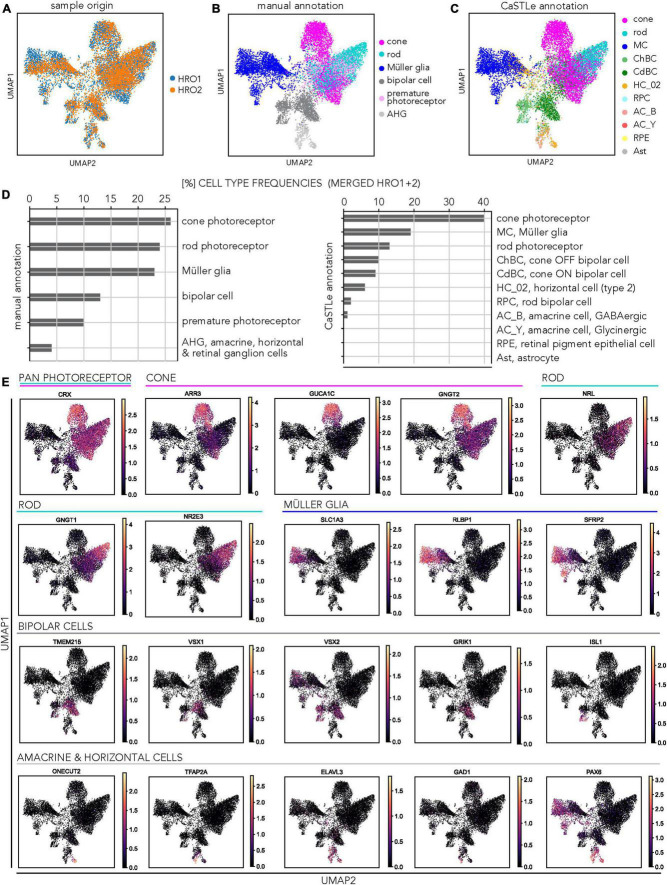
Transcriptomic analysis of CYST-derived human retina organoid cell composition. **(A)** HRO single-cell RNA-seq analysis: UMAP plot shows the low-dimensional embedding of the integrated dataset, including both HRO samples. **(B)** UMAP plot of the integrated HRO dataset, manually annotated for major cell types pseudocolored as indicated. Photoreceptors (light pink): Likely immature cones which could not be clearly assigned. Abbreviations explained in panel **(D)** and Supplementary Table 4. **(C)** UMAP plot showing the cell annotation based on an automatic annotation approach using the transfer learning tool CaSTLe for several retinal cell types pseudocolored as indicated. Abbreviations explained in panel **(D)**. **(D)** Plots depict cell-type frequencies related to manual **(B)** and automatic **(C)** cell annotation. **(E)** Expression pattern of cell type-specific marker genes within the integrated dataset: Expression patterns of selected marker genes used to assign cell-cluster identity across all cells. Retinal cell types are indicated by colored bars. The color gradient corresponds to the log(x + 1) expression of selected marker genes per cell. Data have been generated from D200 HROs of the 5A line. Data have been deposited on Gene Expression Omnibus. Additional data in Supplementary Figure 10.

To assess the cell composition by a different method, we studied single-cell transcriptome datasets of two individual 5A-derived HROs at D200 (HRO1 = 6,665 cells; HRO2 = 5,370 cells). Based on the dataset from HRO1, we previously reported that all major cell types were present, but we had not studied the inner retinal cells in detail. In fact, we had annotated them as bipolars and grouped amacrine, horizontal, and retinal ganglion cells (AHG) together based on manual marker-gene annotation. Here, we added a second HRO to increase cell numbers, analyzed both separately and then integrated both datasets (HRO1 + HRO2 = 12,035 cells). Manual annotation of HRO2 confirmed our previous results with HRO1: About a 1:1:1 ratio of cones (23%), rods (22%), and MG (21%), indicated a cone-richer HRO system. The remaining cells were potential bipolars (15%), AHG (4%), and some photoreceptors (16%) which could not yet clearly be assigned (called premature photoreceptors). To study the inner retinal cell types in more detail, we sought a different approach for cell annotation. Current studies have revealed several subclasses per cell type: 2 horizontal, 12 bipolar, 25 amacrine, and 12 retinal ganglion cell types (Cowan et al., 2020; Yan et al., 2020). For example, bipolar cells are divided into three subclasses: ON and OFF cone bipolars, which release neurotransmitters in response to increases and decreases in illumination of cones, respectively; and rod bipolars, which generate ON responses to stimulation of rods. Manual annotation of cell types in a single-cell transcriptome dataset is subject to expert knowledge, is time consuming, and reproducibility is challenging. As an alternative, various computational algorithms have been developed in recent years that use a reference dataset in which the cell types are already defined to annotate the query dataset. Here, we used an automated annotation tool based on transfer learning called CaSTLe (Lieberman et al., 2018), to complement the manual annotation of our HROs datasets. CaSTLe is based on a random forest architecture that uses informative genes from a reference dataset to classify new datasets: Here, as a reference we used a previously-published single-cell RNAseq dataset by Cowan et al. (2020) of post-mitotic HROs (30- and 38-week-old) that was generated using a protocol different to our CYST protocol and was characterized as rod-richer. Based on this reference dataset (Supplementary Table 4), we were able to annotate eleven different cell types (via the transfer learning approach): Cones, rods, Müller glia, cone ON and OFF bipolar cells, rod bipolar cells, retinal pigment epithelial cells (RPE), GABAergic and glycinergic amacrine cells, horizontal cells, and astrocytes. To analyze the transcriptional similarity across biological replicates, we integrated both HRO samples (Figure 6A). Based on the general sample overlap (Figure 6A), overlap of selected cell types (Supplementary Figure 10A), and the results of the manual (Figure 6B) and automatic annotation approaches (Figure 6C), we observed a significant agreement between both HRO samples. Retinal cell types such as cones, rods and MG form the largest populations of cells with regionally defined clusters, although there were some frequency differences. Separate UMAPs of the two individual HRO datasets (Supplementary Figures 10B, C) displayed comparable trends to the integrated data (Figures 6B, C). Nearly all MG cells were similarly annotated using both the CaSTLe pipeline and manual approach for both HRO1 and HRO2 (Supplementary Figures 10D–F). However, the CaSTLe annotation yielded nearly three times more cones than rods compared to manual annotation (Figure 6D). This could be because some cells classified as rods, and the premature photoreceptor population in the manual annotation, were identified as cones via CaSTLe. Cone photoreceptors revealed robust annotation results in both strategies. Across both organoids, approximately half of the manually annotated rods were annotated as cones using CaSTLe, resulting in a smaller percentage of detected rods. One notable difference between the two HROs was the more pronounced representation of the premature photoreceptor population in HRO2, as indicated by the manual annotation (Supplementary Figure 10B). Nonetheless, the distribution of CaSTLe-annotated rods and cones remained similar between the two HROs (Supplementary Figure 10C). Most interestingly, CaSTLe was able to annotate the inner retinal cell types in more detail: The cluster manually defined as bipolar cells was annotated as three separate cone ON bipolar, cone OFF bipolar, and rod bipolar cells. Similarly, the manually-annotated AHG cell population was divided into GABAergic and glycinergic amacrine cells and horizontal cells. However, no retinal ganglion cell population could be detected, confirming our immunostaining data. All other cell types, like RPE, were either rare or could not be detected. Examples of UMAP embeddings of the integrated HRO dataset shows expression patterns of selected marker genes (Figure 6E) that overlap with the clusters identified by manual and CaSTLe annotation for the related retinal cell types (Supplementary Figures 10E, F), for example: CRX (pan-photoreceptor marker), ARR3 (cones), NRL (rods), RLBP1 (MG), VSX1 and VSX2 (bipolars), and ELAVL3/4 (amacrines and horizontals). Overall, transfer learning-based annotation enabled us to confirm our manual annotation of scRNA-seq data from two HROs, and also presented a higher resolution of annotation including subtypes of inner retinal cells that were previously unidentifiable.

### Maturation of retina organoids derived from the CYST protocol

**FIGURE 7:**
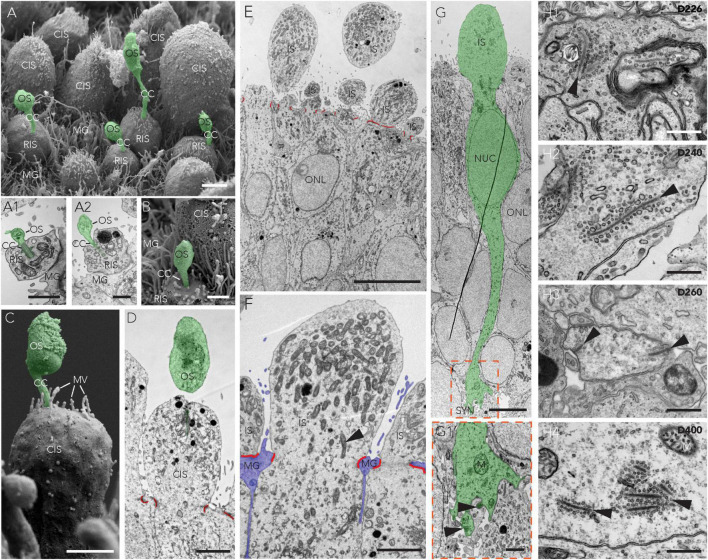
Retinal maturation in CYST-derived human organoids. **(A–H)** Ultrastructural analysis of HROs derived from the 5A line at multiple time points of postmitosis differentiation by transmission and scanning electron microscopy showing the presence of multiple maturation features, including photoreceptor inner (IS) and outer (OS) segments **(A–D)** and an outer limiting membrane **(D–F)** arising from the junctional complexes (red lines) connecting adjacent Müller glia (MG) and photoreceptor cells **(F)**. Mitochondria-rich IS [**(A1,A2,D–F);** arrowhead in panel **(F)** labels one of many mitochondria] and synaptic terminals (**G,G1**; arrowheads) featuring synaptic ribbons (**H1–H4**; arrowheads) were also observed. Cone (CIS) and rod (RIS) inner segments presented with a major difference in size: CISs are much larger than RISs. Furthermore, there was evidence of structures resembling cone and rod nascent outer segments [OS; **(A–D)**]. Scale bars: **(A–C,E,I,J)**, 2 μm; **(C’,F,G)**, 1 μm; **(D)**, 500 nm. CC, connecting cilium; CIS/RIS, cone/rod inner segment; D, day; M, mitochondria; MG, Müller glia; MV, microvilli; NUC, nucleus; ONL, outer nuclear layer; OS, outer segment; SYN, synapse.

To assess photoreceptor maturation based on their specific cellular structure in this HRO system, we performed ultrastructural analysis at various time points after the end of retinogenesis (Figure 4), between D150 and D400, using scanning (SEM, Figures 7A–C) and transmission (TEM, Figures 7A1, A2, D–H) electron microscopy. Analysis in HRO cross-sections by TEM allows investigation of cell ultrastructural features, specifically, subcellular compartments and organelles – like synapses and mitochondria, whereas morphological features of the retinal surface (like PIS/POS) can be evaluated by SEM on HRO wholemounts. Mature human photoreceptors show a characteristic cell morphology (Mustafi et al., 2009; Molday and Moritz, 2015; Goldberg et al., 2016) which we observed in the HRO epithelium: The photoreceptor apical cell processes reach outside the outer retinal surface (Figures 7A, E), these processes frequently each entail the photoreceptor inner segment (PIS). Sometimes a connective cilium connects a PIS to a small/rudimentary outer (POS) segment (Figures 7A1, A2, B–D, F). This is especially apparent in en-face views of whole HROs by SEM, with numerous evenly distributed PISs on the HRO surface (Figures 7A–C). Potential cone and rod PISs could be identified due to their known major difference in size: Cone PISs are much larger than rod PISs (Polyak, 1941). By SEM we also observed connective cilia and rudimentary POSs (D200; Figures 7A–C). However, even at D400 we did not observe any elongated, properly-matured POSs with layered membrane stacks (Mustafi et al., 2009; Molday and Moritz, 2015; Goldberg et al., 2016). Until recently, more mature photoreceptors had not yet been observed, independent of the protocol or hiPSC line used (Nakano et al., 2012; Gonzalez-Cordero et al., 2013; Zhong et al., 2014; Lowe et al., 2016; Wahlin et al., 2017; Capowski et al., 2019). Interestingly, further photoreceptor maturation has been observed in HRO-derived photoreceptors transplanted into the mouse retina *in vivo* (Gasparini et al., 2022) and addition of defined culture supplements may improve POS formation in HROs in culture (West et al., 2022). TEM imaging at the apical border of HRO sections showed junctional complexes connecting adjacent photoreceptor and Müller glia cells (Figures 7D–F), which form the OLM. We further observed that photoreceptors extended from the cell soma, localized in the ONL, a basal process into the OPL: ultrastructural evidence supports the potential formation of synapses. Notably, we find that photoreceptors develop synaptic terminals with synaptic ribbons (Figures 7G, G1) in HROs, as previously observed in human photoreceptors transplanted into the mouse retina (Ribeiro et al., 2021; Gasparini et al., 2022) or in HROs (Wagner et al., 2022). Here, we observed synapses in HROs from D200 up to at least D400 (Figures 7H1–4, arrowheads). However, we do not know yet if these photoreceptors are connected to bipolar neurons like in the functional human retina.

**FIGURE 8:**
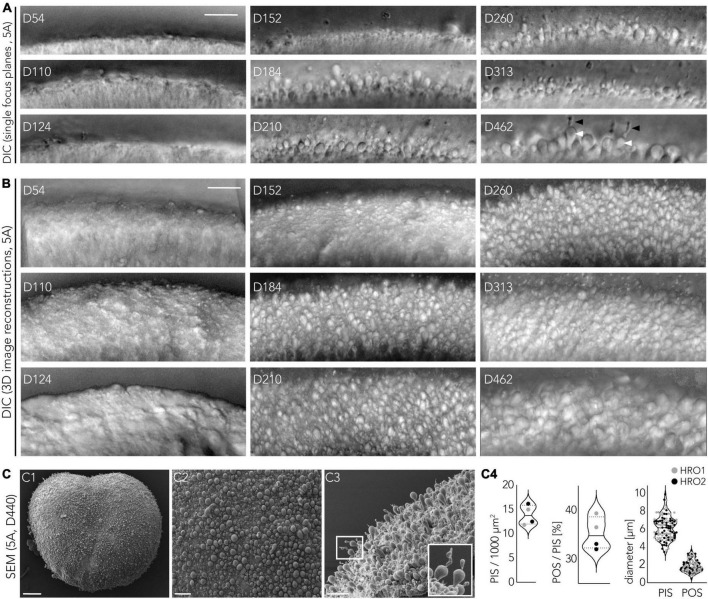
Photoreceptor structural maturation in CYST-derived human retina organoids. **(A,B)** Differential interference contrast (DIC) live imaging of the HRO surface to identify potential emerging photoreceptor inner and outer segments (PIS, POS; two examples are highlighted by white and black arrowheads, respectively). HROs were derived from the 5A line. Images represent single focus planes **(A)** or 3D projections of 100 μm-thick optical stacks **(B)**. **(C)** Ultrastructure analysis by scanning electron microscopy (SEM). Two HROs derived from the 5A hiPSC line were analyzed at D440. Whole organoid **(C1)** and magnified regions of interest images shown from en-face **(C2)** or as side-view **(C3)**. Exemplary PIS and POS are magnified (**C3**, box). Quantitative analysis **(C4)** for photoreceptor (PR) inner and outer segments (PIS, POS) was performed using en-face images **(C2)** (*n* = 2 HROs). The number of total photoreceptors was estimated by counting PISs (two ROIs/n). The presence of POSs was analyzed on 1247–1303 PISs per HRO (544–703 PISs/ROI; two ROIs/n) and the diameters of PISs and POSs were measured (50 PISs and POSs/n). Black and gray dots indicate data from two different HROs. Scale bars: **(A,B,C2–C3)**, 25 μm; **(C1)**, 100 μm. Related data are provided in Supplementary Table 3.

To gain further insight into the timing of PR maturation during HRO development, we performed live imaging using differential interference contrast microscopy (Figures 8A, B; 5A-derived HROs): We observed that cellular protrusions (i.e., PISs) formed on the HRO surface starting at about D150; these covered the HROs homogenously by D180 and remained at least up to D462. At about D200, we infrequently observed additional structures appearing from the PIS-like protrusion, which were potential POSs. To quantitatively assess PISs and POSs, we studied them on SEM images (Figures 8C1–4): We counted (mean ± SD) 14 ± 2 PISs per 1,000 μm^2^ HRO surface area (*n* = 2, D440). About 35 ± 3% (mean ± SD) of PISs showed a POS-like structure at D440 (Figure 7A). The diameter (mean ± SD) of the PISs was 6.2 ± 1.3 μm and of the POSs 1.9 ± 0.7 μm. This is within the range of previous reports on HROs (Völkner et al., 2022) and the primary human retina (Polyak, 1941). Taken together, several parameters indicate that HROs in the CYST protocol not only undergo dynamic development and complete retinogenesis, but also continue to dynamically mature.

## Discussion

In this study, we assessed the reproducibility, efficiency, and variance of a conceptually distinct protocol for HRO generation: Acutely dissociated cell clusters of hiPSCs spontaneously form neuroepithelial cysts that differentiate into an eyefield and, subsequently, a retina. Our main findings are: Comparative studies showed that HRO generation is reproducible from seven hiPSC lines that are derived by different methods and source cells. Organoidogenesis recapitulates several major stages of human retinal development and morphogenesis; retinogenesis is reproducibly completed by D200. Retinal cell composition analysis shows that all hiPSC lines consistently have a rather high proportion of cone photoreceptors than found on average in the human retina (1:20 cone-to-rod ratio; Hussey et al., 2022). These data confirm our previous observation (Völkner et al., 2022) across multiple different hiPSC lines: The CYST protocol provides an HRO system with a cell composition of about 1:1:1 cone, rod, and MG, comparable to the retinal subregion at the interface of the fovea–parafovea of the human macula (Bringmann et al., 2018; Reichenbach and Bringmann, 2020). Further, all other major retinal cell types of the inner retina develop, although retinal ganglion cells are rarely maintained in post-mitotic HROs. Overall, HRO variability seems to correlate more with hiPSC line than with the round of differentiation. Ultrastructural and histological analysis indicate that, based on photoreceptor-cell structure and retinal stratification, HROs may develop and mature to a neonatal-like state. Notably, we demonstrate an approach for systematically comparing organoid protocols side-by-side across different hiPSC lines through parallel differentiation. Our data suggest that, at least in our hands, the CYST protocol might provide a higher efficiency, i.e., a higher HRO yield, quality, and stability, than one of the first HRO protocols that was based on cell aggregate formation from single hiPSCs. However, these results are limited and thus should be considered carefully since we compared both protocols just for some parameters and only with four hiPSC lines, and other hiPSC lines might show different results. Future larger scale systematic protocol comparisons are required to determine difference, which might facilitate organoid protocol optimizations. Together, the presented data advances our understanding and supports the reliability of the CYST-derived HRO system: It provides insights into HRO variances and potential protocol differences; it also specifies parameters, as well as new perspectives for optimizing future organoid systems and their application in basic and medical research.

Since the pioneering studies, several modified protocols for HRO generation have been established (Llonch et al., 2018; Wagstaff et al., 2021). However, many key questions remain for robust HRO generation, effective applications, and comparability between research studies. Several different protocols are currently used by the vision research community for various applications, but the commonalities and differences between protocols have not yet been fully determined. For example, it is still unclear why most HRO systems are rod-photoreceptor dominant, and only some have a higher proportion of cones (Lowe et al., 2016; Kim et al., 2019; Völkner et al., 2022). Our data presented here further support our previous finding that the CYST protocol reliably provides cone-richer HROs. Further, our parallel protocol comparison confirmed differences in cone composition: This approach might provide the chance to determine the underlying mechanisms of differential photoreceptor development. Another example, one HRO system has been optimized for a very high yield at superior quality (Cowan et al., 2020), but so far has been reported to work best with four out of 23 hiPSC line; other established HRO systems have not yet addressed variability at the interorganoid- and experiment-level. Here, we show that the CYST protocol reproducibly generates HROs from seven hiPSC lines, supporting our previous data showing low variance at the interorganoid level (Völkner et al., 2022). Several studies have identified defined stages of retinal development and readout parameters using immunostaining, live imaging, and cell function (Capowski et al., 2019; Kaya et al., 2019; Mellough et al., 2019; Sridhar et al., 2020; Prameela Bharathan et al., 2021), but there are still no effective standardized quality control time points to assess HROs. To further explore this for the CYST protocol, we analyzed two time points during retinogenesis, D120 and D200, which we show can serve to monitor variance during and at the end of retinogenesis, respectively. Bulk and single-cell transcriptome data from human primary retina and HROs are continuously expanding in amount and quality, providing a basis to develop classifications for developmental stages, cell-type composition, and maturation stages (Hoshino et al., 2017; Collin et al., 2019; Kaya et al., 2019; Cowan et al., 2020; Voigt et al., 2020; Gasparini et al., 2022). Transcriptome and other types of analysis of multiple independent experiments and hiPSC lines, or integration of data across protocols and experiments will be informative for protocol optimization, as will studies comparing the primary human retina and HROs (Cowan et al., 2020; Sridhar et al., 2020; Wahle et al., 2023). Taken together, it is still unclear to what extend HROs present differential properties due to protocols, hiPSC lines, experiments, laboratories, and experimenters: There is still no defined global guideline with specific routine readout parameters for effective HRO production. Notably, research communities of other organs have started to establish a consensus, guidelines, and systematic parameters for organoid systems (Marsee et al., 2021; Pasca et al., 2022). These, in combination with defined strategies to systematically compare organoid protocols, would facilitate further optimization of HRO systems with defined properties. To effectively plan future comparative protocol studies, and to further optimize organoid systems, a comprehensive characterization of each HRO protocol across multiple hiPSC lines will be useful. To promote these aims, here we further studied the CYST protocol to provide data across development, and hiPSC lines. Further, we established a potential strategy for side-by-side comparison of HRO protocols.

Our data support earlier reports concluding that different culture protocols share similar retinal developmental stages, suggesting robust reproduction of conserved mechanisms (Nakano et al., 2012; Hoshino et al., 2017; Capowski et al., 2019; Sridhar et al., 2020; Eldred and Reh, 2021). However, there are still multiple differences between various protocols and data available for HRO generation, in particular the choice of source cells, the method for induction of differentiation, cell culture media components modulating early and late stages of retinogenesis, and analysis methods (e.g., cell-specific immunostaining markers and genes to determine cell composition). Here, we extended our studies of the CYST protocol, which involves several unique modifications compared to the pioneering (Meyer et al., 2011; Nakano et al., 2012) and most other protocols (Llonch et al., 2018; Wagstaff et al., 2021; Supplementary Figures 4A, B): Cell differentiation is induced from hiPSCs by the formation of hiPSC cluster-derived neuroepithelial cysts that effectively develop into the eyefield lineage (Zhu et al., 2013), and are induced to differentiate into the retinal lineage by transient adherent culture (Lowe et al., 2016; Völkner et al., 2021b,^2022^). EC23 and serum are supplemented during retinogenesis; the serum is also used for HRO maintenance; and N2 supplement is used during cyst formation and late retinogenesis, as well as for HRO maintenance. Like the early 2D and retinal differentiation protocols, HRO protocols also involve several factors applied at defined time points throughout retinogenesis to potentially support differentiation at several stages (Llonch et al., 2018; Wagstaff et al., 2021). However, the optimal protocol has not yet been identified, some of the underlying developmental mechanisms are still incompletely understood, and further protocols optimized for specific retinal features are continuously being developed (Kaya et al., 2019; Mellough et al., 2019; Pan et al., 2020; Zerti et al., 2020; Wagstaff et al., 2021; Chen et al., 2022; Chew et al., 2022; Sanjurjo-Soriano et al., 2022; Yamasaki et al., 2022). Of note, many pluripotent stem cell-based differentiation protocols rely on the formation of embryoid bodies (EBs), 3D cell aggregates that mimic some features of a developing embryo and that have the ability to produce cells from all three germ layers (Hopfl et al., 2004). The principle of EB formation from a single-cell suspension or cell clusters relies on low attachment substrates that promote cell aggregation. The outcome of EB differentiation is, however, highly dependent on parameters like culture medium and EB size (Messana et al., 2008; Mohr et al., 2010; Meyer et al., 2011; Nakano et al., 2012). In an attempt to harness inherent heterogeneity and achieve consistent differentiation results toward a specific population, including the retina, a fixed number of cells are seeded in U- or V-shaped cell-culture plate wells that promote cell aggregation, while maintaining derived cell aggregates in suspension (Nakano et al., 2012; Kaewkhaw et al., 2015; Wahlin et al., 2017; Hallam et al., 2018). In other labs, 3D retinal differentiation has also been accomplished from cell clumps acutely derived from dissociated pluripotent stem cell (Meyer et al., 2011; Boucherie et al., 2013; Zhong et al., 2014; Mellough et al., 2019). Notably, for most HRO protocols it is still unclear if the hiPSC derived aggregates or cell clumps differentiate via an EB-like state or directly differentiate via the neuroectodermal lineage into retina. While EBs might reproduce part of the natural lineage from pluripotent stem cells to the somatic cells and organ of choice, they also present a source of potential contamination with other, unwanted cell types. This might include other regions of the brain, like the diencephalon, that are part of the natural lineage of retinal development. On the other hand, combined development of cerebral organoids with eye-like structures or with retina organoids as assembloids also might be beneficial to model their connections (Fligor et al., 2021; Gabriel et al., 2021; Ludwig et al., 2023). While some studies in HROs noted the presence of non-retinal or extra-retinal domains of variable size, e.g., other parts of the eye or nervous system, a systematic quantitative analysis has not yet been performed (Browne et al., 2017; Deng et al., 2018; Phillips et al., 2018), and these domains are not always evident in sections that focus on an isolated region of a single organoid. Here, we observed that extra-retinal structures, possibly parts of the ciliary body of the eye, infrequently develop in the AGG and CYST protocols, and this seems to be hiPSC-line dependent. HRO systems might be more effective and homogenous in yield if development of non-retinal cells is prevented.

Our data raise the question of whether protocol modifications that shorten or avoid the EB and neuroectodermal differentiation stages prior to eyefield differentiation might reduce organoid production time, and increase specificity and yield in retina organoid differentiation. Notably, differentiation toward a neuroectodermal and eyefield stage can be effectively achieved within 5 days through the production of 3D neuroepithelial cysts from hiPSC clusters which can be further differentiated into retinal pigment epithelial cells (Zhu et al., 2013) or neural retina (Lowe et al., 2016; Völkner et al., 2021b,^2022^; Gasparini et al., 2022; Koukourakis et al., 2022; Wagner et al., 2022). Neuroepithelial cysts are characterized by a polarized, pseudostratified epithelium surrounding a single lumen (Zhu et al., 2013): Their formation possibly relies on pre-established cell-cell interactions, as cell clusters rather than single cells are used to form 3D structures. In addition, embedding hiPSC clusters directly into pure Matrigel from the beginning (D0) might also play a key role by providing a physical substrate and other cues, whereas in the AGG protocol Matrigel is added later and at a lower concentration. It has been suggested that the interaction between pluripotent cells and an extracellular matrix, rather than the growth factors associated with it, is necessary for the rapid organization into neuroepithelia (Boucherie et al., 2013), by providing signals leading to breaking symmetry and establishing apical-basal polarity (Ranga et al., 2016). In a different approach, Reichman et al. (2017) devised a method to differentiate HROs from confluent hiPSCs without EB formation, and thus also induced cells to differentiate into the eyefield and retinal-cell lineage within a few days. It is still unclear if, in this confluent hiPSC approach (Reichman et al., 2017), random cell clusters also spontaneously form neuroepithelial cyst-like rosettes in 2D that are comparable to the 3D CYST protocol (Zhu et al., 2013). Further, hiPSC-derived clump-based protocols also involve the formation of neuroepithelia, which are considered to be a major determinant for HRO yield (Lowe et al., 2016; Mellough et al., 2019). However, it is still unclear if the onset of neuroepithelial differentiation in hiPSC-derived clumps and other approaches is synchronous, and if its variance determines how effectively, homogenously, and reproducibly retinogenesis proceeds. Here, we observed that within a few days Matrigel-embedded cell clusters self-organized into lumen-containing cysts with neuroretina identity. We hypothesize that the homogeneity of the starting cell population, pre-existing cell-cell interactions, cell culture media composition, and forced contact with Matrigel, could be key for achieving a uniform identity within the cyst, and thus positively influence the efficiency of retinal differentiation. In support of our hypotheses, our preliminary data suggests a higher number of HROs in the CYST compared to the AGG protocol, however, to validate this will require many parallel differentiation experiments. For example, the observed high variance in HRO yield for the 5A line might depend on the quality of cyst formation. Interestingly, we and subsequently others have previously shown that unbiased manual dissection of early-stage neural epithelia into evenly sized portions, called the trisection (Völkner et al., 2019) and checkerboard (Cowan et al., 2020) methods, increase retina organoid yield and quality. These data also possibly support our hypothesis that a homogenous and synchronous starting cell population might be key for efficient HRO production. To help dissect the role of pre-existing cell-cell interactions at the hiPSC level and synchronous differentiation, lineage tracing and tracking of eyefield gene reporters by live imaging could be performed. This would determine if neuroepithelial and eyefield differentiation is highly synchronous at low variance when started from defined cell clusters (CYST protocol). Currently, it is difficult to compare the differentiation efficiency across the available data on protocols and HRO applications, and this will need additional systematic experiments. The parallel differentiation strategy presented here might provide a way to achieve this but will require larger-scale experiments and robust standardized readouts to be conclusive.

Besides the differences between HRO protocols, the hiPSC lines (rather than the round of differentiation) are a well-established source of variability observed in various organoid and cell differentiation studies, even beyond the retina. Our data support this: While some hiPSC lines were able to generate almost exclusively neuroepithelia-containing HROs (e.g., 5A), some were more variable, and one showed a consistently low efficiency (ND5). Here and previously (Völkner et al., 2022), we generated hiPSCs by episomal reprogramming with the goal of maintaining a clinical grade, since the use of lentiviral vectors bears a risk of genotoxicity. Reactivation of residual transgene expression during hiPSC differentiation could indeed affect lineage choice and the functionality of hiPSC derivatives, as recently demonstrated for the RPE cell lineage (Toivonen et al., 2013). The reason for the observed differences between independent experiments is not yet understood, but could be related to the early steps of the protocol, as it is difficult to achieve cell clusters with a uniform number of cells. Furthermore, it has been shown that Matrigel leads to a more heterogeneous colony size, when compared with PEG-based matrices (Ranga et al., 2016). In addition, despite using the same culture and passaging conditions for all hiPSC lines, some showed a higher tendency for spontaneous differentiation (not shown), which could account for some level of heterogeneity. Besides, even though the retinal composition at the end of retinogenesis of neuroepithelia originating from different hiPSC lines were fairly comparable, we observed line-specific differences during development. If differences between hiPSC lines are indeed more pronounced at earlier stages, it would be very interesting to analyze the expression of genes essential for determining neuronal and retinal fate (e.g., OTX2, PAX6, RAX, or VSX2) at the cyst level, and determine if the lines that are shown to be more efficient and perform better (e.g., 5A) have an earlier and perhaps more stable expression of such genes. Interestingly, our data support a previous study reporting that differences between organoids at earlier stages were more related to the hiPSC line, while at later stages the differentiation method is more important (Mellough et al., 2019). However, the previous study was not based on a strategy with parallel hiPSC differentiation, which may introduce variance as discussed below. Further, cortical organoids also seem to show higher variability at earlier stages of development (Yoon et al., 2019). Together, although most current HRO protocols appear to reproduce conserved stages in human retinal development (even across more than 20 different hiPSC lines according to the literature) and seem mostly independent of major sources of variance like hiPSC genetic background, experimenter experience, and culture material lots, the heterogeneity in developmental timing (particularly the onset of eyefield and retinal differentiation), overall organoid growth, and final cell composition is still less well understood at the inter- and intraorganoid level as well as between experiments, hiPSC lines, and protocols.

Several previous studies have shown that HRO systems mostly follow a common developmental sequence resulting in the production of all major retinal cell types. Here, we explored developmental cell proliferation, generation of all major retinal cell types, as well as photoreceptor genesis and maturation in the CYST protocol. We confirmed a very high cone:rod ratio in the HROs (Völkner et al., 2022), which is in contrast to most other HRO protocols, the *in vivo* human peripheral retina, and the mouse retina, which all have a higher proportion of rod photoreceptors. Previously, it was unclear if this depended on the protocol or also on the cell line. Here, we observed that the cone-richer cell composition is highly reproducible across seven hiPSC lines and up to 24 rounds of differentiation (Supplementary Table 3). To the best of our knowledge, only studies starting with the generation of a neuroepithelial cyst so far have achieved a higher proportion of cones (Supplementary Table 1; Lowe et al., 2016; Kim et al., 2019). Notably, the 1:1 ratio between cones and rods, as previously reported in our system (Völkner et al., 2022), resembles some parameters of the parafoveal region of the human retina (Bringmann et al., 2018), although a more detailed comparison is necessary to determine to what extend this represents a defined part of the *in vivo* human retina. The small difference in the cone:rod ratio observed between our own study (1.04–0.63 in HROs from seven hiPSC lines) and that reported by Kim et al. (2019) (1–2.8 depending on the readout) could be explained by the use of different markers: For example, cones were quantified with ARR3 and OPN1LW/MW, and rods with NRL and RHO. In the primary human retina, all cones should be labeled with ARR3, and more than 90% of all cones should express OPN1LW/MW (Ahnelt, 1998). NRL is a transcription factor essential for rod fate determination (Mears et al., 2001) whereas RHO is expressed in mature rods. We and others previously reported that the levels of OPN1LW/MW and RHO expression may still be variable in HROs at D200, and they therefore might not be optimal markers, whereas others, like NRL, ARR3, and CRX, are expressed even in immature photoreceptors. Further, small differences in the cone:rod ratios between different hiPSC lines and protocols could be due to the observed differences in developmental timing. An alternative explanation might be that different hiPSC lines produce different signaling factors and/or different amounts of these factors: That could impact the ratio between retinal cells (Brooks et al., 2019). However, data presented here and our previous analysis of D200 HROs indicated low intra- and interorganoid variance in terms of rod and cone cell numbers (Völkner et al., 2022), and although opsins seemed not to be fully upregulated at that stage, cones and rods each showed distinct, comparable gene expression patterns based on single-cell transcriptome data. Only a small number of photoreceptors in D200 HROs appeared perhaps to be still less mature (Völkner et al., 2022). To assess developmental maturation, longer-term HRO studies will be required.

Additionally, several other parameters have not yet been studied in HROs in detail, like the exact sequence of neurogenesis assessed by gold standard methods like birth dating, as reported for mouse retina organoids (Völkner et al., 2016). Further, while we show by two different methods that inner retinal neurons are present, more detailed analyses are also still required to study cell-subtypes and their variance. Interestingly, although HROs at D200 were comparable in terms of overall organization, layering, and photoreceptor and Müller glia cell composition regardless of the hiPSC line, we observed slight differences in the dynamics of retinogenesis and HRO yield between the hiPSC lines. Here, we show that HROs from all seven hiPSC lines are all post-mitotic at D200, but retinogenesis varies at D120. If retinogenesis ended heterogeneously between D120 and D200, cell maturation might still vary within and between organoids. Our data confirm a previous report showing that temporal organoid staging should be performed carefully, since multiple stages of differentiation can coexist (Capowski et al., 2019). In another study, a screening platform has been developed to measure cell differentiation and overall progression through development based on quantification of specific fluorescent reporters (Vergara et al., 2017). This method presents advantages such as automation and the possibility of scaling-up, and could be used to establish effective readouts to monitor variance in retinogenesis. Given the long-term, laborious nature of organoid culture, establishing a standardized method to evaluate the efficiency of each differentiation round at an early time point would be highly advantageous. Taken together, our data indicate a robust HRO system providing a cone-richer cell composition across several different hiPSC lines, supporting our previous studies showing that this HRO system is useful for several research applications (Völkner et al., 2021b,^2022^; Gasparini et al., 2022; Koukourakis et al., 2022; Wagner et al., 2022).

Although various HRO protocols and applications have been reported, including pathology modeling, the baseline stability of HROs under control condition has not always been studied in detail (Chirco et al., 2021): Some studies have reported an absence (Völkner et al., 2022; West et al., 2022) or presence (Leong et al., 2022) of GFAP or other reactive gliosis markers in post-mitotic HROs, and induction of gliosis upon pathology and prevention by experimental manipulations (Völkner et al., 2022; West et al., 2022). Using the parallel HRO generation strategy, our study suggests that HROs generated by the AGG protocol, at least in our hands, might develop some changes at D200 suggesting spontaneous degeneration: Deficits in retinal structure, and the development of reactive gliosis. In contrast, we did not observe any evidence for spontaneous HRO degeneration in the CYST protocol based on cell numbers, retinal structure, and an absence of GFAP, which confirms our previous data (Völkner et al., 2022). While GFAP is known to be expressed in some astrocytes of the healthy nervous system *in vivo*, like those in the retinal ganglion cell layer (Tao and Zhang, 2014; Luna et al., 2016), Müller glia are thought to express it at very low levels, but upregulate it upon most retinal pathologies, as well known from animal models (Karl et al., 2008; Löffler et al., 2015; Sardar Pasha et al., 2017). Thus, although neuronal cell death at a given time point might appear rare: Monitoring secondary induced reactive gliosis might give a more sensitive readout. However, it is not known whether GFAP is truly absent or low in healthy human retinas, but its upregulation in human patients has frequently been observed (Wu et al., 2003; Bringmann et al., 2006, 2009). We previously showed that GFAP is a readout to monitor pathologic changes in mouse retinas and mouse retina organoids in culture (Sardar Pasha et al., 2017; Völkner et al., 2021a). Notably, it is well known that primary retinas derived from animals spontaneously start to degenerate upon cell culture, which might be due to the change in environment and damage resulting from cutting the optic nerve. In contrast, retina organoids are generated in a cell-culture environment, and spontaneous degeneration has been observed at variable times after the end of retinogenesis (Ito et al., 2017; Völkner et al., 2021a; Leong et al., 2022). In pathology modeling, pathogenesis might be altered by spontaneous degeneration due to insufficient culture or to protocol conditions, in addition to a genetically or otherwise-induced damage or cell stress. For example, studies in mice have shown that low-level retinal damage may have neuroprotective effects (Thiersch et al., 2009). Thus, monitoring and improving the maintenance of HROs in a healthy retina-like state will be key for many applications. Our comparison of hiPSC lines and HRO protocols suggest that both might determine HRO quality and stability. Our observations in AGG-derived HROs are reminiscent of previous observations in organotypic culture of primary retinas (Löffler et al., 2015; Sardar Pasha et al., 2017; Schnichels et al., 2021) and mouse retina organoids (MRO) (Ito et al., 2017; Völkner et al., 2021a; Leong et al., 2022), which each spontaneously start to degenerate immediately and after some extended culture, respectively. Previous studies of several MRO protocols have shown incomplete retinal development and loss of inner retinal cells (Eiraku et al., 2011; Hiler et al., 2015; Ito et al., 2017; Brooks et al., 2019; Decembrini et al., 2020; Völkner et al., 2023). However, it is still unknown if these might cause further degeneration (structural deterioration and onset of gliosis). We observed that although the inner retina is incomplete in MROs, and developmental cell death is ongoing, reactive gliosis and degeneration do not develop immediately, but only after longer-term culture (Völkner et al., 2021a) or experimental stimulation (Völkner et al., 2023), indicating a regulated process and suggesting that this might depend on the maturation stage or other additional changes. Notably, developmental cell death also does not induce reactive gliosis *in vivo* (Mervin and Stone, 2002; Löffler et al., 2015; Sardar Pasha et al., 2017; Völkner et al., 2021a). Since retinal ganglion cell are born but not maintained until end of retinogenesis in HROs derived by both protocols, comparable to previous reports by others, this might not explain the upregulation of reactive gliosis in the AGG but not CYST protocol. It is still not known to what extend such spontaneous retinal pathologies in culture are caused by selected cell-culture or other protocol parameters, whether these reproduce any defined pathologic changes in retinal damage and disease *in vivo*, or whether the mechanisms underlying degeneration of primary retinas and retina organoids in culture are related. Further, in our recent work (Völkner et al., 2022), we observed that a genetic program might be activated in MG upon stimulation with HBEGF and TNF: These have previously been shown to regulate defined neuroprotective and neurotoxic programs of reactive gliosis in other types of glia. Whether human MG in primary human retinas or experimental models, like HROs, may provide distinct beneficial and detrimental functions under homeostatic physiological conditions has not yet been shown. It still needs to be determined, if spontaneous GFAP upregulation indicates a reactive gliosis response in AGG-derived HROs at D200, and if so, whether it provides beneficial or detrimental functions. Thus, like for most current MRO protocols, our study raises the question to what extend HROs in the protocols currently available maintain longer-term stability in culture.

It might be particularly interesting to learn if recent HRO protocol optimizations, like those supporting progression in photoreceptor maturation (West et al., 2022), also facilitate further overall HRO maturation and longer-term stability. This is highly relevant for most research studies on retinal physiology, pathology, and therapy. For example, experimental pathology modeling in MROs has shown that it is possible to experimentally induce photoreceptor degeneration, but not to reliably study it for extended timeframes due to additional spontaneous retinal degeneration (Ito et al., 2017; Völkner et al., 2021a). In comparison, the CYST protocol provides experimental access and reliable experimentally-induced pathology modeling in HROs up to D250 (Völkner et al., 2022). However, it is still unclear if complete pathogenesis of gene mutation induced, specifically, slower-developing or later-onset diseases, could also be modeled reliably in HROs without additional spontaneous degeneration. Further, animal studies have indicated that gene therapy success might depend on the pathologic state of the retina (Cideciyan et al., 2013), and thus variable retinal stability within and between organoids would limit comparative analysis of viral vector transduction efficiency (Völkner et al., 2021b). HRO-derived cells have been used for cell replacement therapy studies (Llonch et al., 2018; Gasparini et al., 2022; Wagner et al., 2022), but it is not yet known if cell integration and survival might be impaired or more variable if donor cells are derived from spontaneously degenerating organoids. Our side-by-side HRO differentiation strategy presented here might not only be useful to assess other protocols, but upon protocol modification also to generate organoids with different properties from the same batches of hiPSCs in one experiment. For various applications, like comparative studies of pathology models and therapy development, it might in the future be useful to establish two robust HRO systems from the same hiPSC batch in parallel that are either rod or cone dominant, or provide some other distinct anatomical and functional features of the peripheral, central, or macular region of the human retina. Taken together, it might be possible to develop differential HRO systems with distinct retinal properties, cell components, and long-term stability to support different research applications: To achieve this a comprehensive characterization of the current HRO systems will be key. With this goal, our study provides further insight into the CYST-protocol and a potential strategy to determine HRO protocol differences, and advances our understanding for future protocol optimization.

## Data availability statement

The datasets presented in this study can be found in online repositories. The names of the repository/repositories and accession number(s) can be found below: https://www.ncbi.nlm.nih.gov/geo/, GSE174215; https://www.ncbi.nlm.nih.gov/geo/, GSE237007.

## Ethics statement

All experiments involving hiPSCs were performed in accordance with the Ethical Standards of the Institutional and/or National Research Committee, as well as with the 1964 Helsinki Declaration and its later amendments, and approved by the Ethical Committee at the Technische Universität Dresden. This research study is part of a project that has been approved by the Ethics Committee of the TU Dresden (EK390102017). The newly-generated CRTD3 (hPSCreg: CRTDi003-B1) hiPSC line and several previously published or commercially available ones (CRTD1, CRTD2, 5A, ND5, GBE, and IMR90) were used in this study (Table 1). The CRTD3 hiPSC line was generated from CD34- positive cells isolated from peripheral blood of consenting healthy donors (Ethics Committee of Technische Universität Dresden, EK 363112012).

## Author contributions

MC, MV, and MK: conceptualization. MC, MV, FW, TK, LS, ND, SU, SW, AN, CG, SK, BS, MS, KZ, SC, JH, and MK: investigation. MC, MV, FW, LS, and MK: writing—original draft. MC, MV, FW, LS, MA, TK, and MK: writing—review and editing. JH and MK: supervision. MA, TK, JH, and MK: funding acquisition. All authors contributed to the article and approved the submitted version.

## Footnotes

1
https://hpscreg.eu/cell-line/CRTDi003-B

2
allencell.org/cell-catalog
