# Supplementary material for: Reliability of human retina organoid generation from hiPSC-derived neuroepithelial cysts
Source: Front Cell Neurosci. 2023 Oct 6;17:1166641. doi: 10.3389/fncel.2023.1166641 (PMC10587494; doi:10.3389/fncel.2023.1166641)
Supplement: Supplementary file 1 [file Table_1.DOCX]

Supplementary Table 1

| Cell of Interest | | Marker | Amount | Time point | Protocol | Quantification Method | Reference |
| --- | --- | --- | --- | --- | --- | --- | --- |
| **Photo-receptors** | CRX | | 15% | D90 | AGG | CRXp-GFP H9 line; FACS; n=3; >1000 cells/n; L=1 | 1 |
|  |  |  | 12-18% | D126 | AGG | CRX::Venus hESC line; IHC; n=4; >1000cells/n; L=1 | 2 |
|  |  |  | 69% | D135 | CLUMP | IHC of dissociated cells from CRX^+/tdTomato^+ OV; CRX+ cells; n=3; L=1 | 3 |
|  |  |  | 4% | D30 | CYST | IHC; CRX+ cells/DAPI+ cells; N=3/L; n=16-27/L; L=2 (calculated mean) (Fig.4C) | this study |
|  |  |  | 19% | D60 | CYST | IHC; CRX+ cells/DAPI+ cells; N=3/L; n=16-27/L; L=2 (calculated mean) (Fig.4C) | this study |
|  |  |  | 28% | D90 | CYST | IHC; CRX+ cells/DAPI+ cells; N=3/L; n=16-27/L; L=2 (calculated mean) (Fig.4C) | this study |
|  |  |  | 33% | D120 | CYST | IHC; CRX+ cells/DAPI+ cells; N=1-5/L; n=7-23/L; L=5 (calculated mean) (Fig.4C) | this study |
|  |  |  | 41% | D200 | CYST | IHC; CRX+ cells/DAPI+ cells; N=2-3/L; n=9-28/L; L=7 (calculated mean) (Fig.4E) | this study |
| **Cones** | ARR3 | | 12% | D90 | AGG | IHC; ARR3+ cells/DAPI+ cells; N=3; L=1 | 1 |
|  |  |  | ~20% of ONL | ≥D120 (W17) | CLUMP | IHC; ARR3+ cells/ONL; n≥6/L; L=3 | 4 |
|  |  |  | 18% | D140 (W20) | CLUMP | IHC; ARR3+ cells/image; N=3, n=30 images; L=1 | 5 |
|  |  |  | 25% | D200 | CYST | IHC; ARR3+ cells/DAPI+ cells; N=2/L; n=10/L; L=2 (calculated mean for 5A line) | 8 (Fig.1c) |
|  |  |  | 23% | D200 | CYST | IHC; ARR3+ cells/DAPI+ cells; N=13, ≥5n/N, L=4 (calculated mean for 5A, CRTD1, CRTD2, IMR90) | 8 (Fig.S11) |
|  |  |  | 38% | D200 | CYST | Flow cytometry; ARR3+ cells, L=2; N=1-2/L; 2-3 samples/N (calculated mean) | 8 (Fig.2e) |
|  |  |  | 18% | D200 | CYST | IHC; ARR3+ cells/DAPI+ cells; N=2-7/L; n=22-50/L; L=7 (calculated mean) (Fig.5C) | this study |
|  |  |  | 15% | D200 | AGG | IHC; ARR3+ cells/DAPI+ cells; N=2, n=7, L=1 (5A) (Fig.3C) | this study |
|  | OPN1SW | | 9% | D135 | CLUMP | IHC of dissociated cells from CRX^+/tdTomato^+ OV; OPN1SW+ cells; n=3; L=1 | 3 |
|  | THRB | | 17% | D135 | CLUMP | IHC of dissociated cells from CRX^+/tdTomato^+ OV; THRß + cells; n=3; L=1 | 3 |
|  | OPN1LW/MW | | 176 cells | D215 | CYST | whole mount IHC; 6 stacks of optical sections; n=4 (calculated mean); L=1 | 6 |
|  | Top 20 markers/cluster | | 34% | D240 | CYST | single cell RNA-seq; L=1 | 6 |
|  | Top 20 markers/cluster | | 28% | D200 | CYST | single cell RNA-seq; L=1 | 8 (Fig.1g) |
|  | manual annotation  (Suppl. Table 4) | | 26% | D200 | CYST | single cell RNA-seq; Manual annotation; L=1; (integrated data HRO1 + HRO2) | this study |
|  | CaSTLE annotation (Suppl. Table 4) | | 40% | D200 | CYST | single cell RNA-seq; CaSTLe annotation; L=1; (integrated data HRO1 + HRO2) | this study |
| **Rods** | NRL | | 23% | D90 | AGG | IHC; NRL+ cells/DAPI+ cells; N=3; L=1 | 1 |
|  |  |  | ~80% of ONL | ≥D120 (W17) | CLUMP | IHC; NRL+ cells/ONL; n≥6/L; L=3 | 4 |
|  |  |  | 33% | D135 | CLUMP | IHC of dissociated cells from CRX^+/tdTomato^+ OV; NRL+ cells; n=3; L=1 | 3 |
|  |  |  | 25% | D200 | CYST | IHC; NRL+ cells/DAPI+ cells; N=2/L; n=10/L; L=2 (calculated mean) | 8 (Fig.1c) |
|  |  |  | 21% | D200 | CYST | IHC; NRL+ cells/DAPI+ cells; N=2-4; n=13-50/L; L=7 (calculated mean) (Fig.5C) | this study |
|  |  |  | 22% | D200 | AGG | IHC; NRL+ cells/DAPI+ cells; N=2, n=7, L=1 (5A) (Fig.3C) | this study |
|  | RHO | | 118 cells | D215 | CYST | whole mount IHC; 6 stacks of optical sections; n=4 (calculated mean); L=1 | 6 |
|  | RCVRN+ARR3− | | 26% | D200 | CYST | Flow cytometry; RCVRN+ARR3− cells, L=2; N=1-2/L; 2-3 samples/N (calculated mean) | 8 (Fig.2e) |
|  | Top 20 markers/cluster | | 25% | D240 | CYST | single cell RNA-seq; L=1 | 6 |
|  | manual annotation | | 24% | D200 | CYST | single cell RNA-seq; Manual annotation; L=1; (integrated data HRO1 + HRO2) | this study |
|  | CaSTLE annotation | | 13% | D200 | CYST | single cell RNA-seq; CaSTLe annotation; L=1; (integrated data HRO1 + HRO2) | this study |
|  | Top 20 markers/cluster | | 25% | D200 | CYST | single cell RNA-seq; L=1 | 8 (Fig.1g) |
| **Cone:Rod ratio** | ARR3:NRL | | 1:2 | D90 | AGG |  | 1 |
|  | ARR3:NRL | | 1:4 | ≥D120 | CLUMP |  | 4 |
|  | RXRγ:NRL | | 1:4 | D126 | AGG | IHC; n=4; >200cells/n | 2 |
|  | (OPN1SW+THRß):NRL | | 1:1 | D135 | CLUMP |  | 3 |
|  | OPN1LW/MW: RHO | | 1:1 | D215 | CYST | IHC and single cell RNA-seq | 6 |
|  | ARR3:NRL | | 1:1 | D200 | CYST | IHC; N=2/L; n=10/L; L=2 (calculated mean) & single cell RNA-seq; L=1 | 8 (Fig.1c) |
|  | ARR3:NRL | | 1:1 | D200 | CYST | IHC; ARR3+/DAPI+ cells; N=13, ≥5n/N, n>65, L=4 (mean for 5A, CRTD1, CRTD2, IMR90) | 8 (Fig.S11) |
|  | Top 20 markers/cluster | | 1:1 | D200 | CYST | single cell RNA-seq; L=1 | 8 (Fig.2e) |
|  | ARR3:NRL | | 1.1:1 | D200 | CYST | IHC; ARR3+ and NRL+ /DAPI+ cells; calculated mean of all hiPSC lines, N≥24, n≥184 L=7 (Fig.5E) | this study |
|  | ARR3:NRL | | 0.7:1 | D200 | AGG | IHC; ARR3+ and NRL+ /DAPI+ cells; N=2, n=7, L=1 (5A) (Fig.3C) | this study |
| **Müller Glia** | SOX9 | | 24% | D150 | CYST | IHC; SOX9+ cells/DAPI+ cells; N=1-2/L; n=5-10/L; L=2 (calculated mean) | 8 |
|  | SOX9 | | 25% | D200 | CYST | IHC; SOX9+ cells/DAPI+ cells; N=2/L; n=10/L; L=2 (calculated mean) | 8 |
|  | SOX9 | | 26% | D250 | CYST | IHC; SOX9+ cells/DAPI+ cells; N=2/L; n=10/L; L=2 (calculated mean) | 8 |
|  | SOX9 | | 23% | D200 | CYST | Flow cytometry; SOX9+ cells, L=2; N=1-2/L; 2-3 samples/N (calculated mean) | 8 |
|  | SOX9 | | 28% | D200 | CYST | IHC; SOX9+ cells/DAPI+ cells; N=2-4/L; n=16-50/L; L=7 (calculated mean) (Fig.5C) | this study |
|  | SOX9 | | 28% | D200 | AGG | IHC; ARR3+ and NRL+ /DAPI+ cells; N=2, n=7, L=1 (5A) (Fig.3C) | this study |
|  | Top 20 markers/cluster | | 25% | D200 | CYST | single cell RNA-seq; L=1 | 8 |
|  | Top 10 markers/cluster | | 53% | D200 | CLUMP | single cell RNA-seq; L=1 | 7 |
|  | Top 20 markers/cluster | | 25% | D240 | CYST | single cell RNA-seq; L=1 | 6 |
|  | SLC1A3, RLBP1, SFRP2 | | 24% | D200 | CYST | single cell RNA-seq; Manual annotation; L=1; (integrated data HRO1 + HRO2) | this study |

References:

1. Kaewkhaw, R. et al, “Transcriptome dynamics of developing photoreceptors in three-dimensional retina cultures recapitulates temporal sequence of human cone and rod differentiation revealing cell surface markers and gene networks”, Stem Cells, 2015
2. Nakano, T. et al, “Self-formation of optic cups and storable stratified neural retina from human ESCs”, Cell 2012
3. Phillips, M. J. et al. Blood-derived human iPS cells generate optic vesicle-like structures with the capacity to form retinal laminae and develop synapses. *Invest. Ophthalmol. Vis. Sci.* **53**, 2007–2019 (2012).
4. Capowski, E. et al “Reproducibility and staging of 3D human retinal organoids across multiple pluripotent cell lines”, Human Development, 2019
5. Gonzalez-Cordero, A. et al, “Recapitulation of Human Retinal Development from Human Pluripotent Stem Cells Generates Transplantable Populations of Cone Photoreceptors”, Stem Cell Reports, 2017
6. Kim, S. et al, “Generation, transcriptome profiling and functional validation of cone-rich human retinal organoids”, PNAS, 2019
7. Collin, J. et al, “Deconstructing retinal organoids: single cell RNA-seq reveals the cellular components of human pluripotent stem cell-derived retina”, Stem Cells, 2018
8. Völkner, M. et al, “HBEGF-TNF induce a complex outer retinal pathology with photoreceptor cell extrusion in human organoids”, Nat Commun, 2022
