# Supplementary material for: Reliability of human retina organoid generation from hiPSC-derived neuroepithelial cysts
Source: Front Cell Neurosci. 2023 Oct 6;17:1166641. doi: 10.3389/fncel.2023.1166641 (PMC10587494; doi:10.3389/fncel.2023.1166641)
Supplement: Supplementary file 2 [file Table_2.DOCX]

| Antigen | Target | Host | Cat. no. | Vendor | Dilution | Antigen retrieval |
| --- | --- | --- | --- | --- | --- | --- |
| ACTA2 (SMA) | Mesoderm, used for 3-germ layer immunocytochemistry | mouse | A25531 | Thermo Scientific | Applied according to manufacturer’s instructions | |
| ARR1 | Rod photoreceptors | rabbit | PA1-731 | Thermo Scientific | 1:300 | - |
| ARR3 | Cone photoreceptors | mouse | - | Gift from P.R. MacLeish, U Texas, Houston | 1:100 | - |
|  |  | goat | NBP1-37003 | Novus Biologicals | 1:250 | - |
| BRN3 | RGC | goat | sc-31984 | Santa Cruz | 1:400 | Citrate |
| CRX | Post mitotic photoreceptors | rabbit | - | Gift from E. Tanaka, CRTD, Dresden | 1:1000 | DNAse |
| ELAVL3/4 | Developing RGC and AC (early marker), HC | mouse | A-21271 | Invitrogen | 1:300 | - |
| GFAP | Astrocytes, MG end feet (healthy) and cell body (reactive) | rat | 13-0300 | Invitrogen | 1:250 | - |
| MITO | Mitochondria | mouse | MAB1273 | Millipore | 1:200 | - |
| NRL | Rod photoreceptors | goat | AF2945 | R&D | 1:200 | DNAse |
| OCT3/4 (coupled to Alexa Fluor 488) | Used for pluripotency analysis by flow cytometry | mouse | 560253 | BD Pharmingen | Applied according to manufacturer’s instructions | |
| PAX6 | Progenitors, MG, HC, AC, RGC | rabbit | 901301 | BioLegend | 1:500 | DNAse |
| PHOSPHO- HISTONE 3 (PHH3) | Mitotic cells | rat | NB600-1168 | Novus Biologicals | 1:4000 | - |
| PRPH2 | Photoreceptor outer segment | rabbit | 18109-1-AP | Proteintech | 1:100 | - |
| RAX | Eye field marker | rabbit | - | Gift from E. Tanaka, CRTD, Dresden | 1:1000 | DNAse |
| RBPMS | RGC | rabbit | ab152101 | Abcam | 1:1000 | Citrate |
| RCVRN | Photoreceptors, some BC, some RGC | rabbit | AB5585 | Millipore | 1:1000 | - |
| RHO | Rod photoreceptors | mouse | O4886 | Sigma | 1:1000 | - |
| RLBP1 | MG | mouse | MA1-813 | Thermo Scientific | 1:200 |  |
| SOX2 | Progenitors, AC, MG | goat | AF2018 | R&D | 1:200 | DNAse |
| SOX2  (coupled to PE) | Used for pluripotency analysis by flow cytometry | mouse | 560291 | BD Pharmingen | Applied according to manufacturer’s instructions | |
| SOX9 | Progenitors, MG | rabbit | HPA001758 | Sigma | 1:200 | Citrate |
| SOX17 | Endoderm, used for 3-germ layer immunocytochemistry | mouse | ab84990 | Abcam | 1:200 | - |
| SSEA-4  (coupled to V450) | Used for pluripotency analysis by flow cytometry | mouse | 561156 | BD Pharmingen | Applied according to manufacturer’s instructions | |
| TUBB3 (TUJ1) | Ectoderm, used for 3-germ layer immunocytochemistry | rabbit | A25532 | Thermo Scientific | Applied according to manufacturer’s instructions | |
| TRA-1-60 (coupled to Alexa Fluor 647) | Used for pluripotency analysis by flow cytometry | mouse | 560850 | BD Pharmingen | Applied according to manufacturer’s instructions | |
| VSX2 | Progenitors, BC, MG | goat | sc-21692 | Santa Cruz | 1:200 | DNAse |
|  |  | sheep | X1180P | Exalpha | 1:200 | Citrate |

Supplementary Table 2
