# Supplementary material for: Reliability of human retina organoid generation from hiPSC-derived neuroepithelial cysts
Source: Front Cell Neurosci. 2023 Oct 6;17:1166641. doi: 10.3389/fncel.2023.1166641 (PMC10587494; doi:10.3389/fncel.2023.1166641)
Supplement: Supplementary file 7 [file Data_Sheet_1.docx]

Supplementary Material

# Supplementary Figures and Tables

## Supplementary Figures

**Supplementary Figure 1: Characterization of a newly-generated hiPSC line.**

Data supplements Figure 1. Newly-generated CRTD3 hiPSC line was derived from CD34+ peripheral blood cells reprogrammed using CytoTune-iPS 2.0 Sendai Reprogramming Kit.

A: CRTD3 hiPSCs express high levels of pluripotency markers SOX2, OCT4, TRA-1-60, and SSEA4 as determined by flow cytometry. This was confirmed by qRT-PCR for key pluripotency markers according to the International Stem Cell Initiative (ISCI), using the human ES cell Primer Array (not shown).

B: G-banding karyogram of CRTD3 hiPSC shows a normal karyotype.

C: Representative phase-contrast image of CRTD3 hiPSC in culture.

D: Spontaneous trilineage differentiation of CRTD3 hiPSC was confirmed via immunostaining by markers for ectoderm (TUBB3), mesoderm (ACTA2), and endoderm (SOX17), as well as by qRT-PCR using the human ES cell Primer Array (not shown).

Scale bars: 50µm.

**Supplementary Figure 2: Live-imaging microscopy of developing cysts.**

A-B: Representative images from live-imaging movies on cyst formation from fluorescent reporter hiPSC clusters. Imaging was started at differentiation day (D) 0, imaging frequency was one frame per hour.

A: Representative images from Supplementary Movie 1: Live imaging of cyst formation from ACTB-mEGFP reporter hiPSC clusters.

B: Representative images from Supplementary Movie 2: Live imaging of cyst formation from TJP1-mEGFP reporter hiPSC clusters.

Scale bars: 25µm.

**Supplementary Figure 3: Efficiency in HRO generation across multiple hiPSC lines.**

A-B: Data supplement Figure 1.

A: Representative brightfield images of HROs generated using the CYST protocol using multiple hiPSC lines, at different development time points (D30-D120).

B: To distinguish HROs derived by the different hiPSC lines from non-retinal organoids, cryosections were immunostained with anti-RAX antibody. Related analysis is presented in D.

C: To show that RAX labels cells across the retina, cells were immunostained in combination with ARR3 (cytoplasmic cone photoreceptor marker) and SOX2 (nuclear Müller glia marker). HRO was derived from the 5A line.

D: Additional quantitative data plots supplementing Figure 1D. Total number of 3D structures (T3D) at the end time point (D200) divided by the number of starting hiPSC wells (of a 6-well plate). To distinguish HROs from non-retinal organoids, cryosections were immunostained with anti-RAX antibody. Fraction of RAX+ retinal epithelium (%RE): percentage of retinal epithelium in each organoid at the end time point measured as the RAX+ area in relation to total cell area as a proxy. Statistical analysis results (1-way ANOVA, Tukey post-hoc test) are given as follows: The horizontal lines and asterisks (*) above the scatter plots indicate a statistically significant (p<0.05) difference between a selected (color-matched) hiPSC line and other hiPSC lines.

Scale bars: A, B, 500µm; C, 50µm. Related data are provided in Supplementary Table 3.

**Supplementary Figure 4: Two conceptionally different HRO protocols used in this study.**

A-B: Data supplements Figure 2.

A: Schematic representation of the two protocols used in this study to generate human retina organoids (HROs): Cluster-derived neuroepithelial cyst (CYST); and single cell-derived aggregate (AGG) protocol.

B: Schematic representation of key components of the cell-culture medium.

**Supplementary Figure 5: Data supplements Figure 2.**

A-C: Data plots supplement Figure 2.

A: Total number of 3D structures (T3D) at the end time point divided by the number of starting hiPSC wells (6-well plate).

B: Total number of HROs per T3D.

C: HRO yield: Number of HROs (#HRO) at the end time point per starting hiPSC well.

D-F: To distinguish HROs from non-retinal organoids, cryosections were immunostained with anti-RAX antibody.

D: Fraction of RAX+ retinal epithelium (%RE): percentage of retinal epithelium in each organoid at the end time point measured as the RAX+ area in relation to total cell area as a proxy.

E: Representative raw and binary images of cryosections immunostained for RAX and DAPI showing non-retinal 3D tissues with almost no RAX+ cells.

F: Quantification of HROs with extra-retinal structures (see Material and methods section) and two exemplary brightfield images of HROs with extra-retinal structures. Red dotted line highlights extra-retinal structures; arrowhead points to retinal epithelia.

G: Assessment of inner retinal cell types in developing HROs derived by the CYST and AGG protocols: Representative microscopic images of HRO sections immunostained for markers of inner retinal cell types: BRN3 and RBPMS (retinal ganglion cells), and ELAVL3 and ELAV4 (ELAVL3/4; amacrine and horizontal cells, and some retinal ganglion cells). DAPI labels cell nuclei. D, day of differentiation.

Scale bars: E, F, 200µm; G, 50µm. Statistical analysis results (2-way ANOVA) are given as follows (N=2-4 / hiPSC line; in (D): n=3-14/N): * p<0.01. End time points were D200 (5A line) or D90-D100 (CRTD1, CRTD2, CRTD3 lines). Related data are provided in Supplementary Table 3.

**Supplementary Figure 6: Additional data on retinogenesis dynamics in the CYST-derived human retina organoid system.**

Data supplements Figure 4: Progenitor cell proliferation and photoreceptor generation were investigated in selected hiPSC lines and at selected days (D) of development.

A: Supplementary data related to Figure 4 at D200. Cell proliferation was analyzed by immunostaining for the mitosis marker phospho-histone H3 (PHH3) and cell nuclei (DAPI). Representative cryosection images of PHH3-immunostained HROs as used to obtain quantifications shown in Figure 4C, E.

B-D: Supplementary data for HROs at D120 derived from selected hiPSC lines.

B: Photoreceptor genesis was studied by immunostaining for CRX: CRX+ cells were counted per region of interest (ROI) in HRO cryosections and normalized to the number of DAPI+ cells. Representative microscopic images of HRO cryosections derived from the hiPSC lines indicated, immunostained for PHH3 and CRX; related to the quantifications shown in D.

C: Cell proliferation was analyzed by immunostaining for PHH3 and cell nuclei (DAPI).

D: Quantitative analysis based on data as presented in B-C. Each dot in the plots represents the mean of two ROIs per HRO (n=7-23 HROs/N, N=1-4 experiments/line). Cell proliferation was assessed on HRO sections by counting cells immuno-positive for PHH3, normalized to total DAPI area of the same section. Statistical analysis results are given as follows: The horizontal lines and asterisks (*) indicate a significant difference between a selected (color-matched) hiPSC line and other hiPSC lines. Note that the CRX and PHH3 datasets from D120 for the 5A and GBE lines are also shown in Figure 4C.

Scale bars: A, C, 500µm; B, 20µm. A-C: Statistical analysis results (2-way ANOVA, Tukey post-hoc test) are given as follows: * p<0.05. Not all statistical data are shown. Related data are provided in Supplementary Table 3.

**Supplementary Figure 7: Additional quantitative data plots supplementing Figure 5.**

Ratio of the different cell types indicated, and mean coefficient of variation (COV) for cones (C), rods (R), and Müller glia (MG) based on the markers ARR3, NRL, and SOX9, respectively, for each experiment (n=15-50 HROs/N, N=2-7 experiments per line).

**Supplementary Figure 8: Analysis of selected retinal cell types in HROs derived from different hiPSC lines.**

To compare the presence of major classes of inner retinal cells, selected markers were analyzed in HROs derived by the CYST protocol at differentiation day (D) 200. HROs from seven hiPSC lines were assessed: CRTD1, CRTD2, CRTD3, ND5, GBE, IMR90 and 5A; data for the 5A line are shown in Figure 3A. Representative microscopic images are shown of HRO sections immunostained for set 1: BRN3 and RBPMS (retinal ganglion cell markers), as well as ELAVL3 and ELAV4 (ELAVL3/4; marker for amacrine and horizontal cells, and some retinal ganglion cells); and for set 2: PAX6 (marker for amacrine and horizontal cells, and some retinal ganglion cells), VSX2 (bipolar cell marker), and RLBP1 (Müller glia marker). Scale bars: 50µm.

**Supplementary Figure 9: Analysis of selected inner retinal cell types throughout HRO genesis.**

To determine the generation and maintenance of retinal ganglion cells in HROs generated by the CYST protocol, selected markers were analyzed at several timepoints (D, day of differentiation). Representative microscopic images of HRO sections immunostained for BRN3 and RBPMS (retinal ganglion cell markers), as well as ELAVL3 and ELAV4 (ELAVL3/4; marker for amacrine and horizontal cells, and some retinal ganglion cells) are shown. DAPI labels cell nuclei. Scale bar: 50µm.

**Supplementary Figure 10: Additional data for transcriptomic analysis.**

A: Related to Figure 6A. HRO single-cell RNA-seq analysis: Dotplot shows first two principal components of cones, rods, and Müller glia as identified using the automatic annotation workflow across HRO samples.

B-D: Related to Figure 6B-D. UMAP-plots of integrated datasets from both HROs either annotated manually (B) or using an automatic approach using CaSTLe (C) for major cell types pseudocolored as indicated. Bar graphs depict related cell-type frequencies. UMAP plot shown for HRO1 (top left) was previously published in Völkner et al. 2022. D: Alluvial plots for the two different cell annotation approaches shown in (B) and (C) for each of the two HRO datasets.

E-F: UMAP plots highlighting each individual cell type based on manual (E) and CaSTLe annotation in the merge data of HRO1 and HRO2 shown also in combination in Figure 6A.

## Supplementary Tables

**Supplementary Table 1: Quantification of cells of interest in this and other studies.**

Overview of quantification of cells of interest in HROs performed in this and other studies, including protocol, time point analyzed and quantification method. Few protocols are based on the formation of neuroepithelial cysts (CYST) from small hiPSC clusters as an initial step. In contrast, other protocols are based on the aggregation of a defined number of dissociated single cells (AGG) or on small cell clumps directly derived by dissociation of hiPSCs (CLUMP). Of note, this is only one aspect of differ­ences between protocols besides composition of culture medium, addition of supplements and others. Acronyms for differentiation protocols starting from distinct pluripotent stem cell preparations: AGG, differentiation protocol starting by aggregation of a defined number of dissociated single cells; CLUMP, differentiation protocol starting from small cell clumps; CYST, differentiation protocol starting from small cell cluster forming epithelia cysts; HRO, human retina organoid; OV, optic vesicles; ONL, outer nuclear layer; N, independent experiments; n, number of HROs; L, number of hiPSC line; IHC, immunohistochemistry.

**Supplementary Table 2: List of primary antibodies used in this study.**

Cat. no., catalogue number; AC, amacrine cells; BC, bipolar cells; HC, horizontal cells; MG, Müller glia; RGC, retinal ganglion cells.

**Supplementary Table 3: Quantitative and statistical data related to main figures.**

Worksheet names indicate related figures and contain related data, e.g., results (mean ± SD) per figure, sample size, and overview of statistical test. Statistically significant results are depicted in green. N, independent experiments; n, number of HROs; AGG, aggregate protocol; CYST, cyst protocol; See Material and methods section for definition and analysis of: T3D, % RE, and HRO yield.

**Supplementary Table 4: Compression of the high-resolution cell types for the transfer learning in single cell RNAseq analysis.**

## Worksheet 1: To increase the training performance of CaSTLE, a transfer learning approach, the resolution of retinal cell types was reduced (columns A-B) compared to the original dataset (columns C-D) by Cowan et al. 2020.

## Worksheets 2-4: Cell composition analysis data for HRO1 and HRO2, and for integration of both.

## Supplementary Movies

**Supplementary Movie 1: Live imaging of cyst formation from cell clusters of an ACTB-mEGFP reporter hiPSC line.** Imaging was started at differentiation day 0, imaging frequency was one frame per hour. Scale bar: 25µm.

**Supplementary Movie 2: Live imaging of cyst formation from cell clusters of a TJP1-mEGFP reporter hiPSC line.** Imaging was started at differentiation day 0, imaging frequency was one frame per hour. Scale bar: 25µm.
