# Supplementary figures and images for: Reliability of human retina organoid generation from hiPSC-derived neuroepithelial cysts
Source: Front Cell Neurosci. 2023 Oct 6;17:1166641. doi: 10.3389/fncel.2023.1166641 (PMC10587494; doi:10.3389/fncel.2023.1166641)

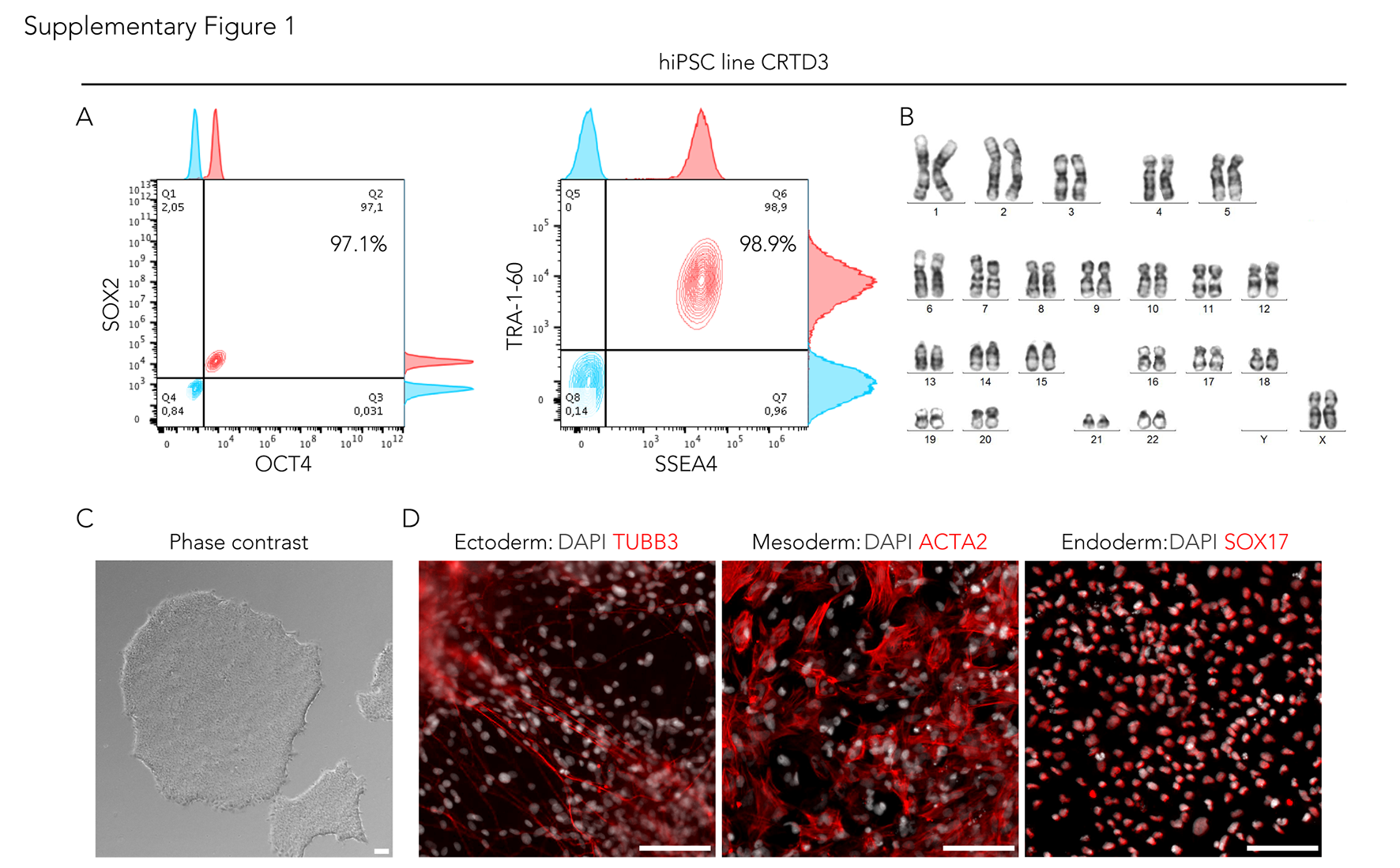

Supplement: Supplementary file 8 [file Image_1.TIF]

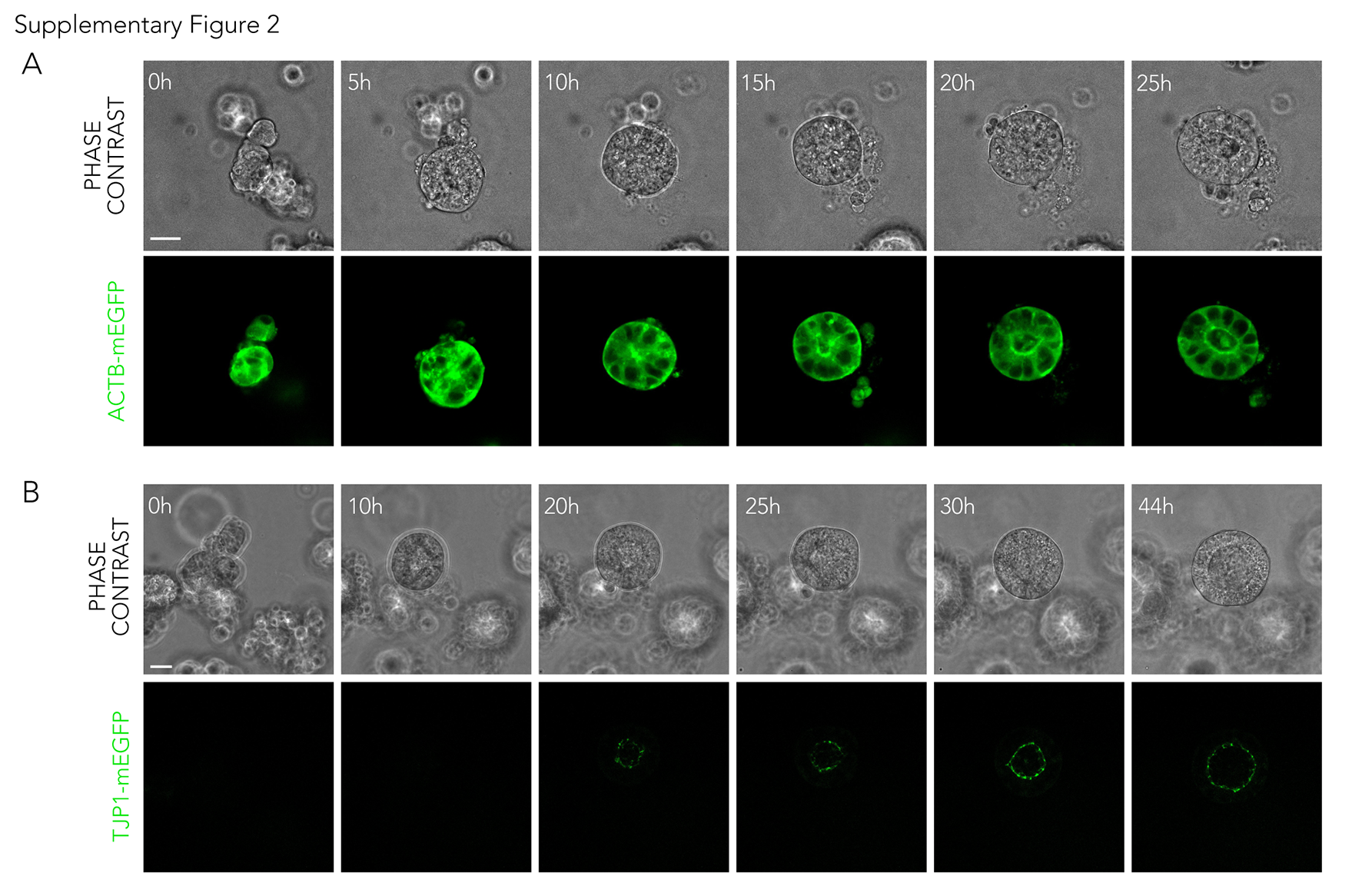

Supplement: Supplementary file 9 [file Image_2.TIF]

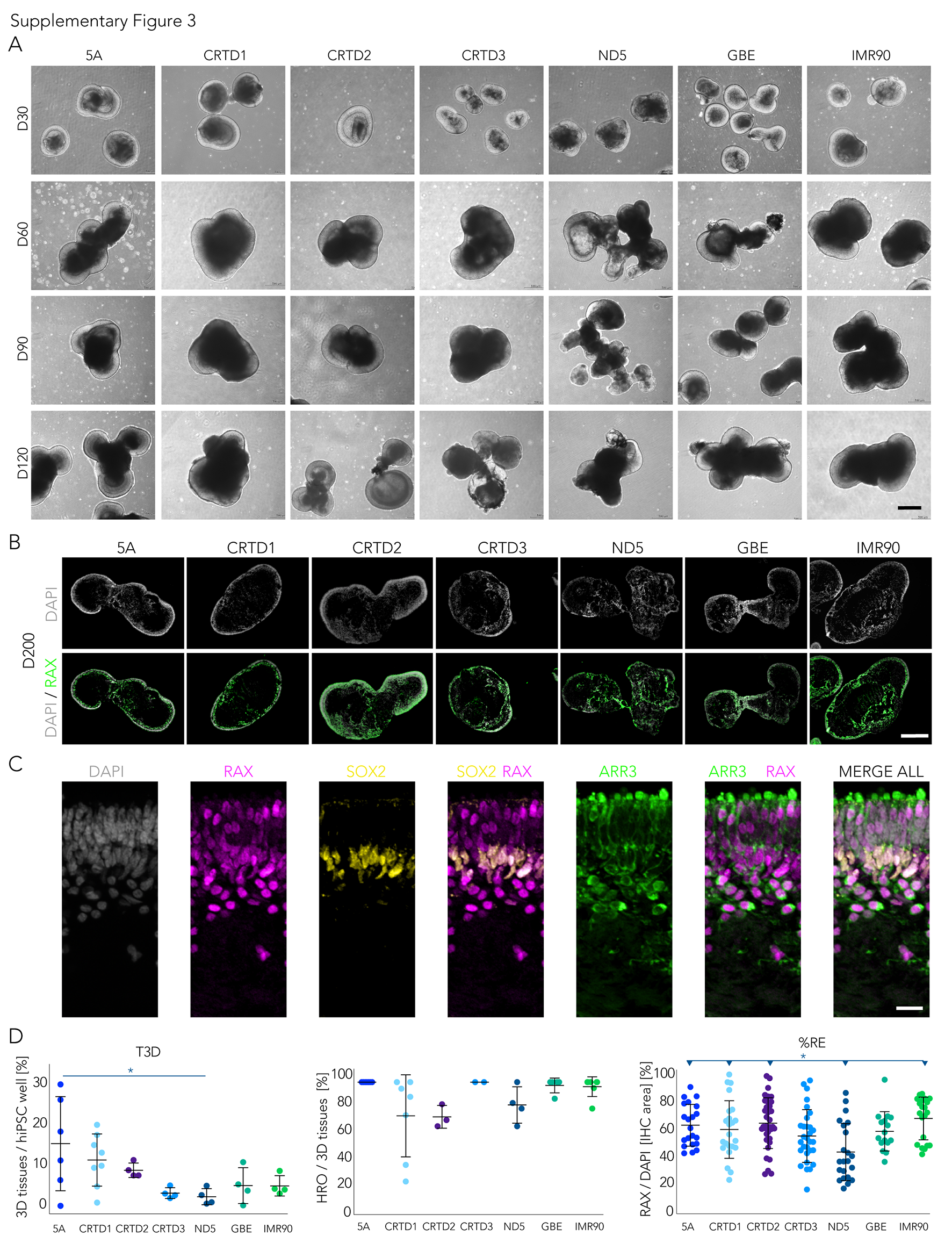

Supplement: Supplementary file 10 [file Image_3.TIF]

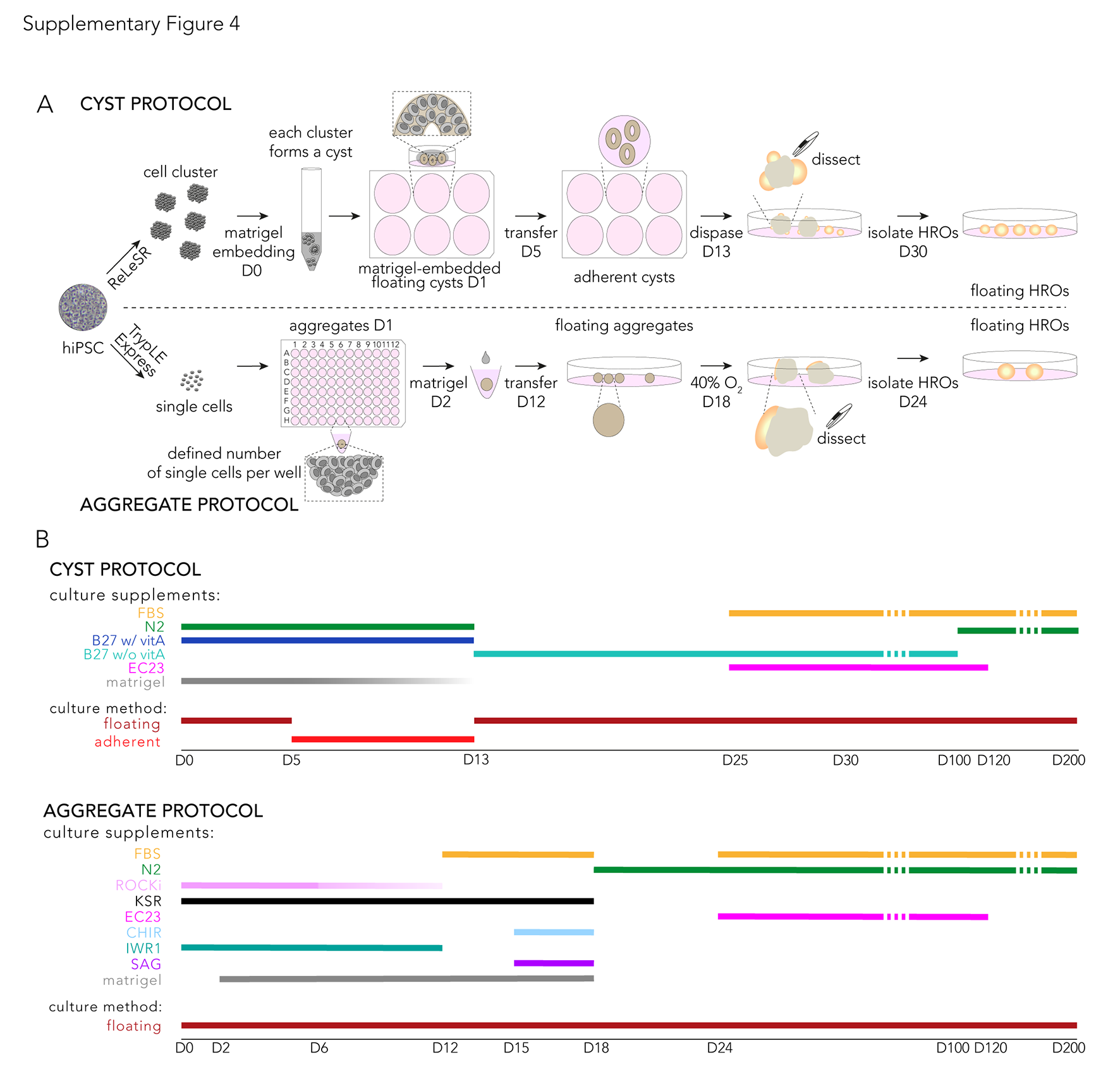

Supplement: Supplementary file 11 [file Image_4.TIF]

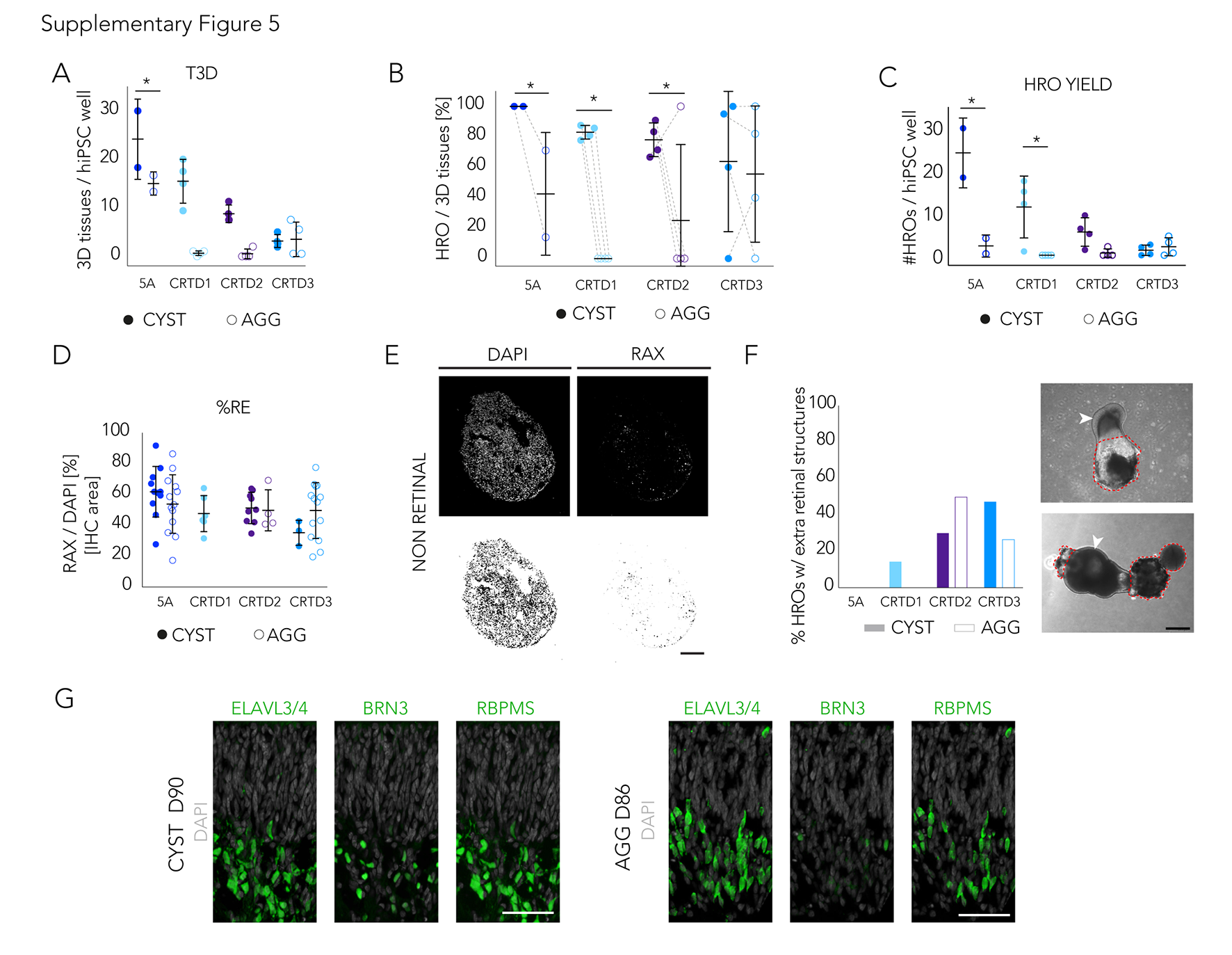

Supplement: Supplementary file 12 [file Image_5.TIF]

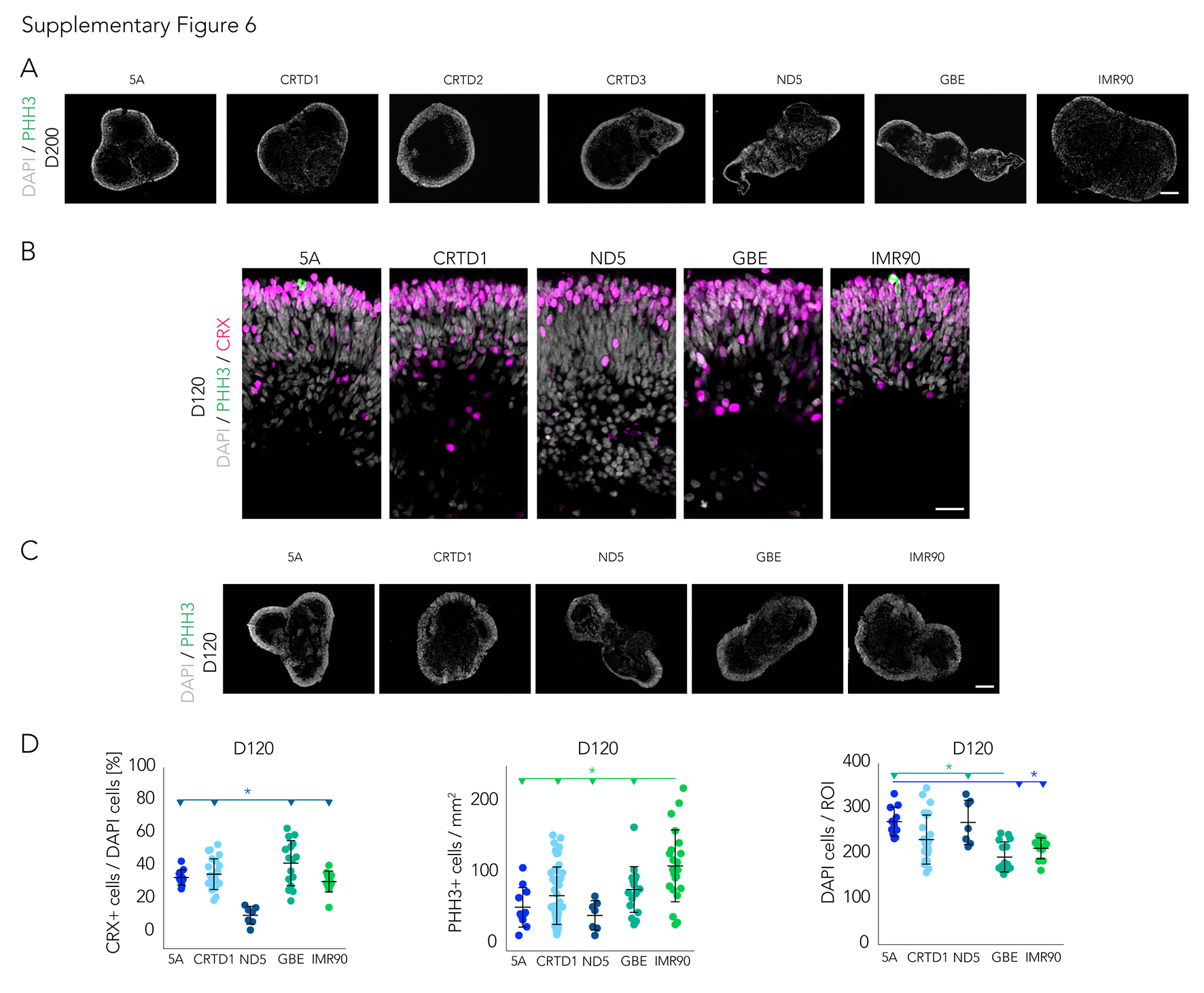

Supplement: Supplementary file 13 [file Image_6.TIF]

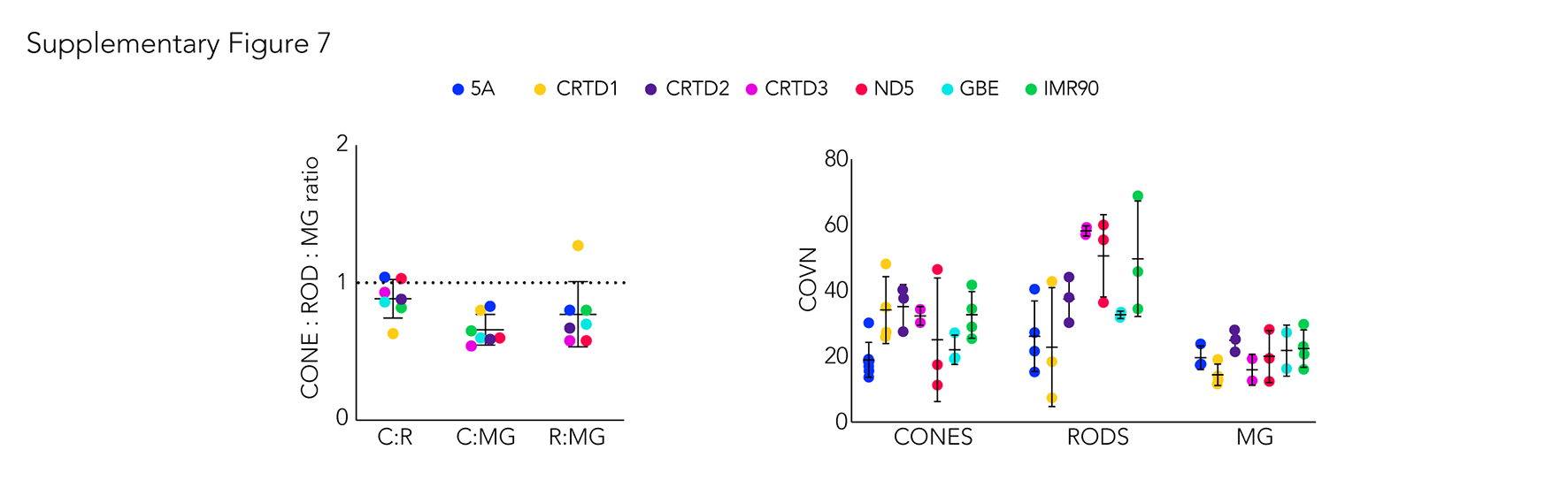

Supplement: Supplementary file 14 [file Image_7.TIF]

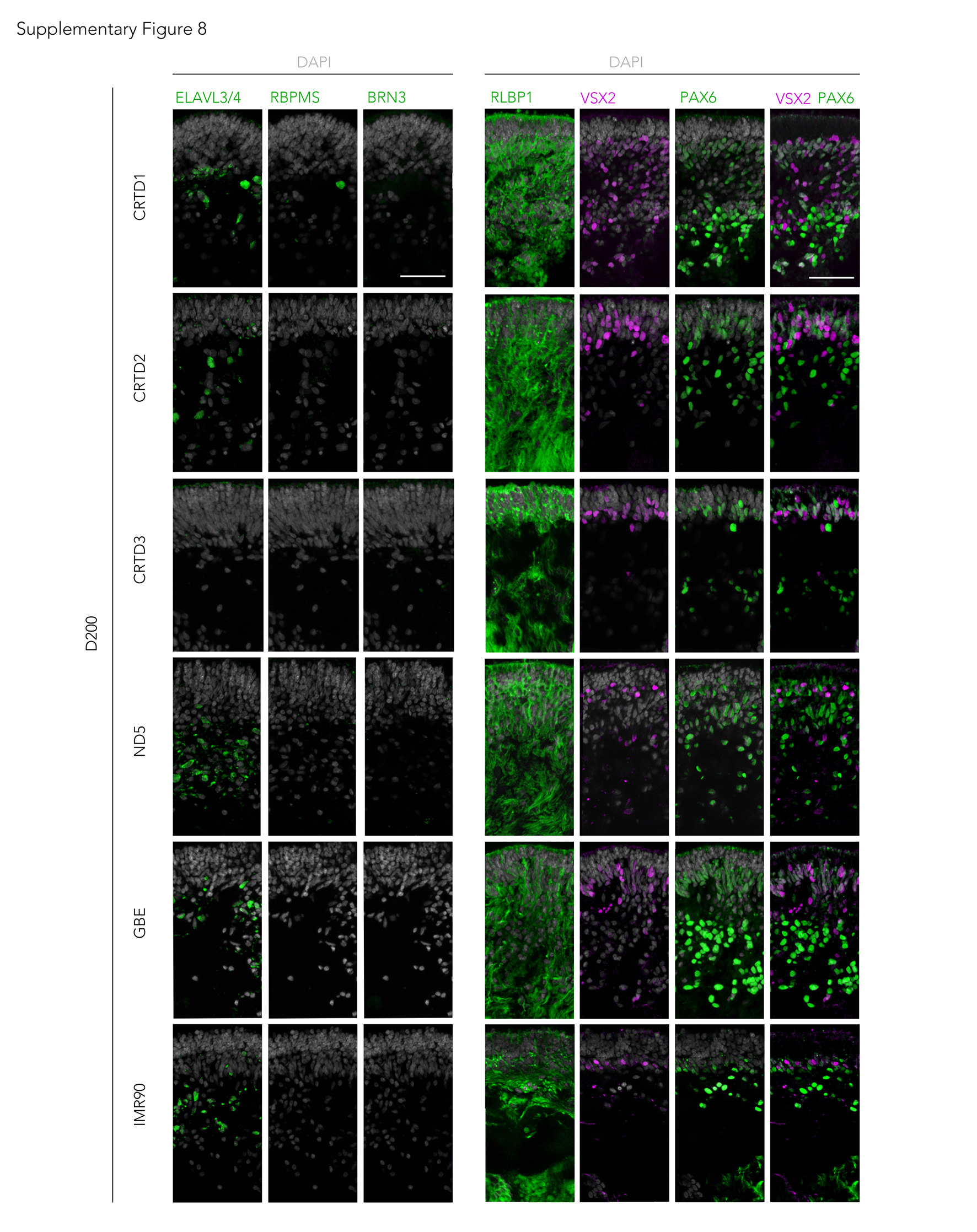

Supplement: Supplementary file 15 [file Image_8.TIF]

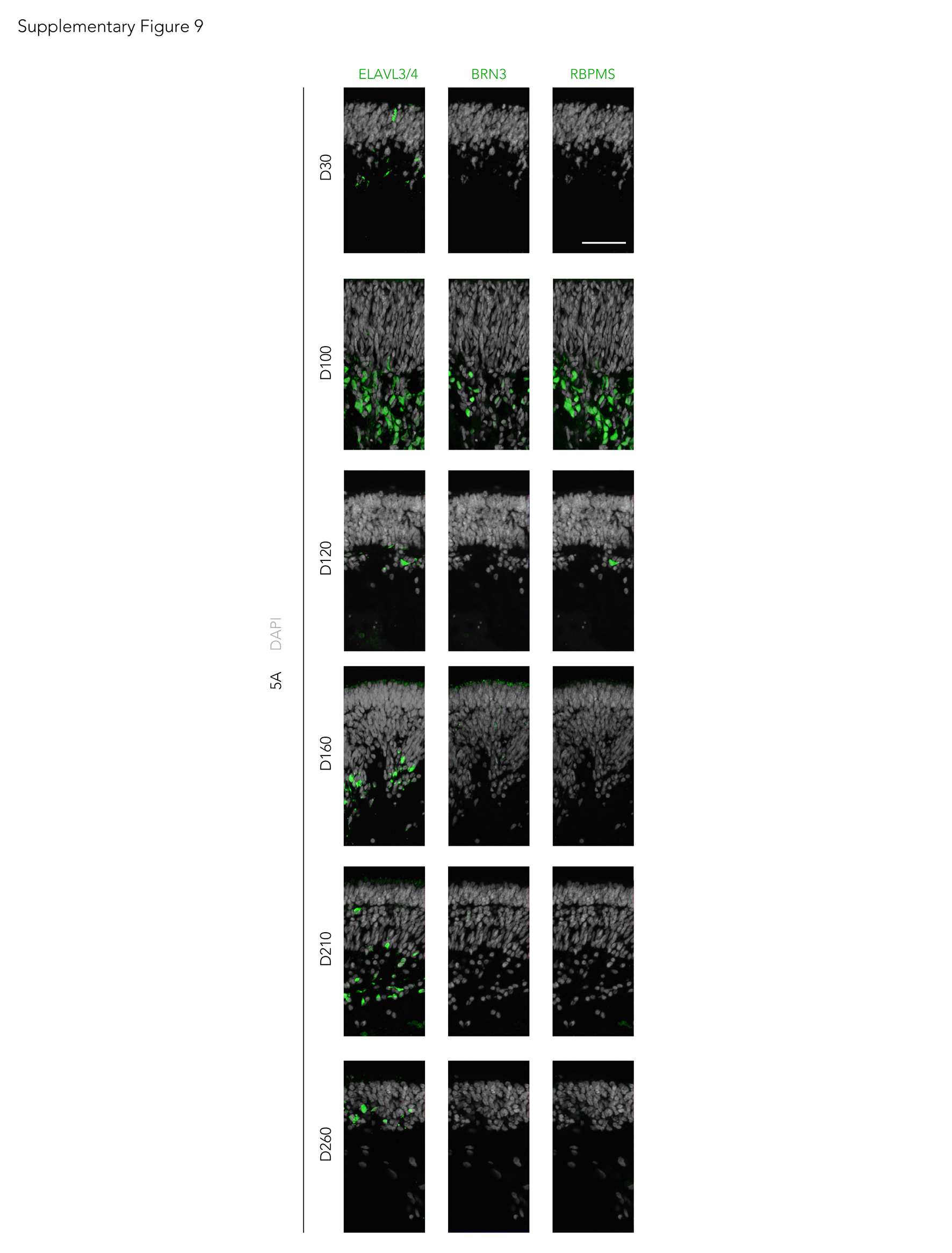

Supplement: Supplementary file 16 [file Image_9.TIF]

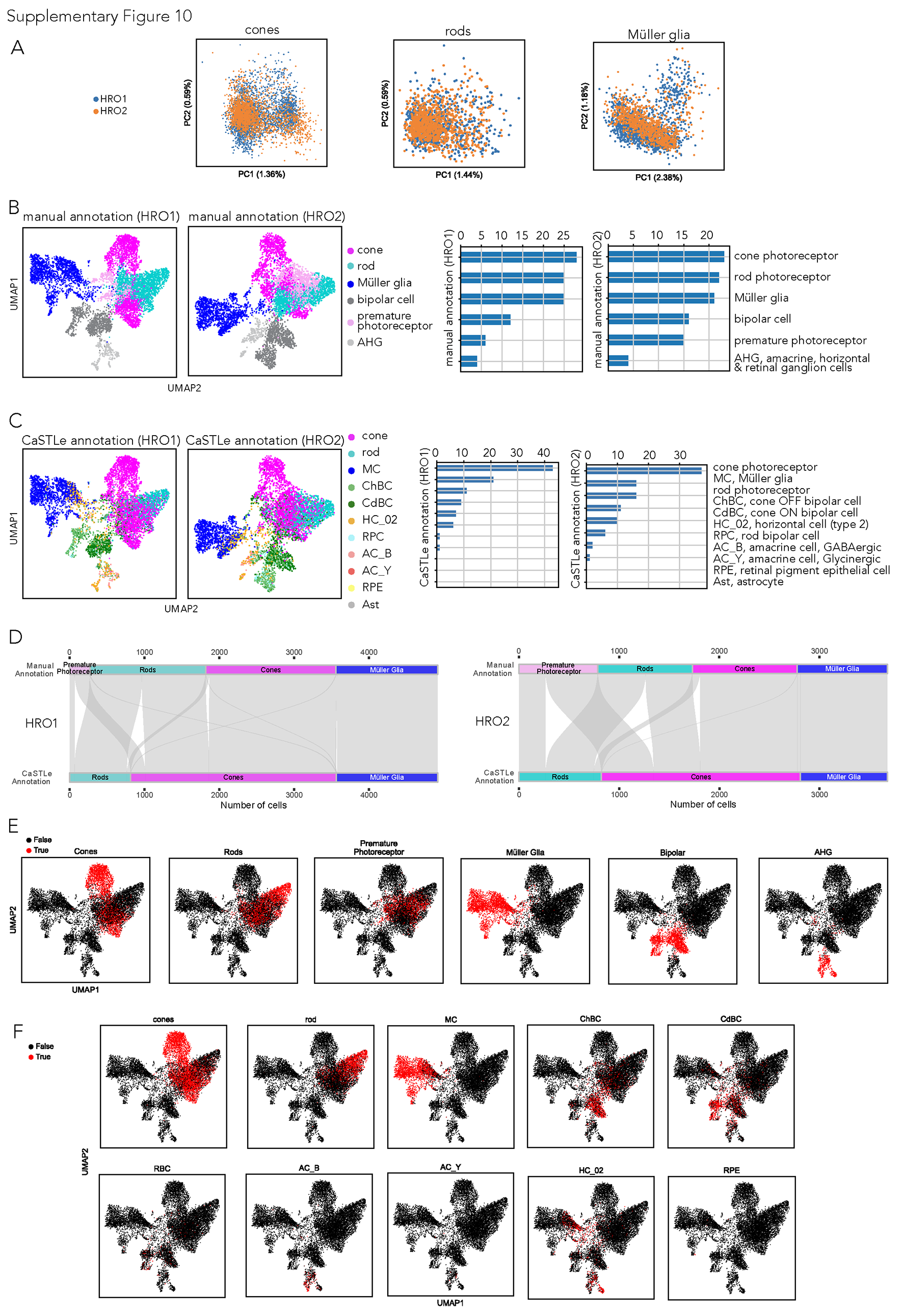

Supplement: Supplementary file 17 [file Image_10.TIF]
